# Crosstalk between BRCA-Fanconi anemia and mismatch repair pathways prevents MSH2-dependent aberrant DNA damage responses

**DOI:** 10.15252/embj.201387530

**Published:** 2014-06-26

**Authors:** Min Peng, Jenny Xie, Anna Ucher, Janet Stavnezer, Sharon B Cantor

**Affiliations:** 1 Department of Cancer Biology, University of Massachusetts Medical School, Women's Cancers Program, UMASS Memorial Cancer CenterWorcester, MA, USA; 2 Department of Microbiology and Physiological Systems, University of Massachusetts Medical School, Women's Cancers Program, UMASS Memorial Cancer CenterWorcester, MA, USA

**Keywords:** Fanconi anemia, FANCJ, mismatch repair, MLH1, replication stress

## Abstract

Several proteins in the BRCA-Fanconi anemia (FA) pathway, such as FANCJ, BRCA1, and FANCD2, interact with mismatch repair (MMR) pathway factors, but the significance of this link remains unknown. Unlike the BRCA-FA pathway, the MMR pathway is not essential for cells to survive toxic DNA interstrand crosslinks (ICLs), although MMR proteins bind ICLs and other DNA structures that form at stalled replication forks. We hypothesized that MMR proteins corrupt ICL repair in cells that lack crosstalk between BRCA-FA and MMR pathways. Here, we show that ICL sensitivity of cells lacking the interaction between FANCJ and the MMR protein MLH1 is suppressed by depletion of the upstream mismatch recognition factor MSH2. MSH2 depletion suppresses an aberrant DNA damage response, restores cell cycle progression, and promotes ICL resistance through a Rad18-dependent mechanism. MSH2 depletion also suppresses ICL sensitivity in cells deficient for BRCA1 or FANCD2, but not FANCA. Rescue by Msh2 loss was confirmed in Fancd2-null primary mouse cells. Thus, we propose that regulation of MSH2-dependent DNA damage response underlies the importance of interactions between BRCA-FA and MMR pathways.

## Introduction

DNA interstrand crosslinks (ICLs) induce a range of cellular responses, including recruitment of DNA repair proteins to the lesion and/or a stalled replication fork. Subsequent processing of ICLs and restart of replication forks require the coordination of several repair pathways, including homologous recombination (HR) and the error-prone DNA damage tolerance mechanism, translesion synthesis (TLS; Sale, 2012). Cells derived from Fanconi anemia (FA) patients or BRCA1/2-associated tumors that lack the BRCA-FA pathway (BRCA-FA cells) are extremely sensitive to agents such as mitomycin C (MMC) that induce ICLs (Moldovan & D'Andrea, 2009; Muniandy *et al*, 2010). This interstrand crosslink (ICL) sensitivity and associated chromosomal aberrations are key determinants to diagnosing genetic deficiency in the BRCA-FA pathway, which has up to 16 components (Sharma & Canman, 2012).

The ICL sensitivity in BRCA-FA cells has been attributed to defects in the repair of intermediates of ICL processing such as DNA double-strand breaks (DSBs; Moldovan & D'Andrea, 2009). In particular, loss of the BRCA-FA proteins BRCA1 and FANCD2 leads to defects in recombination-directed repair. This has been attributed to non-homologous end-joining (NHEJ) proteins that occupy the ends of broken DNA and interfere with DNA end-processing required for HR (Bunting & Nussenzweig, 2010; Aly & Ganesan, 2011). In FANCD2-deficient cells, the NHEJ protein DNA-PKcs is aberrantly phosphorylated (Adamo *et al*, 2010). Furthermore, in BRCA1-deficient cells, HR is restored by elimination of the NHEJ factor, 53BP1 (Bouwman *et al*, 2010; Bunting *et al*, 2010; Aly & Ganesan, 2011). Remarkably, loss of 53BP1 also overcomes early embryonic lethality in BRCA1-nullizigous mice (Cao *et al*, 2009; Bouwman *et al*, 2010; Bunting *et al*, 2012), suggesting that 53BP1 underlies the proliferation defect in BRCA1 mice.

While elimination of NHEJ can normalize growth and HR defects in BRCA-FA cells, ICL repair is not fully restored. For example, in Brca1-null mouse cells, eliminating NHEJ restored HR, but did not fully restore ICL resistance (Bunting *et al*, 2012). Moreover, in Fancd2-null mouse cells, ICL sensitivity was enhanced by inactivation of NHEJ and mice had more severe developmental defects (Houghtaling *et al*, 2005; Bunting *et al*, 2012). Furthermore, in worms that are mutant for the FANCJ (BACH1/BRIP1) homologue, *dog-1*, ICL sensitivity was not suppressed by eliminating NHEJ (Adamo *et al*, 2010). Failure to restore ICL repair in BRCA-FA cells by suppression of NHEJ suggests that the BRCA-FA pathway has additional roles besides suppression of NHEJ. Other functions include protecting replication forks from degradation by nucleases (Schlacher *et al*, 2011, 2012) and orchestrating replication restart through HR, TLS, and other post-replication repair pathways (Kim & D'Andrea, 2012).

The functional relevance is not fully understood; however, several reports have linked the BRCA-FA pathway with proteins of the mismatch repair (MMR) pathway. In particular, BRCA1, FANCD2, SLX4/FANCP, and FANCJ interactions with MMR proteins have been reported (Wang *et al*, 2000; Svendsen *et al*, 2009; Kratz *et al*, 2010; Liu *et al*, 2010; O'Donnell & Durocher, 2010; Shereda *et al*, 2010; Smogorzewska *et al*, 2010; Yoshikiyo *et al*, 2010; Huang *et al*, 2011; Williams *et al*, 2011; Ward *et al*, 2012; Peng *et al*, 2007). Moreover, we found that FANCJ binding to the MMR protein MLH1 is essential for ICL repair (Peng *et al*, 2007; Cantor & Xie, 2010; Xie *et al*, 2010). Further suggesting a functional connection between these pathways, MMR proteins activate the BRCA-FA pathway, including the promotion of FANCD2 monoubiquitination (Huang *et al*, 2011; Williams *et al*, 2011) and also localize FANCJ to sites of ICLs and DNA crosslinks induced by ultraviolet light (Suhasini *et al*, 2013; Guillemette *et al*, 2014).

Proteins of the MMR pathway bind DNA lesions/perturbations through either the heterodimer MutSβ (composed of MSH2 and MSH3) or MutSα (composed of MSH2 and MSH6), which subsequently recruit the MutLα complex (composed of MLH1 and PMS2; Duckett *et al*, 1996; Brown *et al*, 1997; Yamada *et al*, 1997; Zhang *et al*, 2002; Wu & Vasquez, 2008). When processing certain DNA lesions, such as ICLs, MMR complexes from bacteria to human cells have been associated with break induction and promoting apoptosis (Fram *et al*, 1985; Nowosielska & Marinus, 2005; Zhang *et al*, 2007; Fink *et al*, 1996; Nehme *et al*, 1997). This DNA damage response could result from MMR proteins binding ICLs or other DNA structures that form at stalled DNA replication forks. MMR proteins also have genome surveillance functions that counteract error-prone bypass pathways essential for ICL processing (Jiricny, 2006; Sharma & Canman, 2012).

Here, we considered the hypothesis that MMR-dependent responses in the absence of coordination with the BRCA-FA pathway are detrimental and contribute to defects in FA cells. To interrogate the contribution of MMR to ICL processing defects, we eliminated MMR. We found defects were suppressed by loss of MSH2. MSH2 depletion does not appear to enhance DNA repair, but rather attenuates the DNA damage response that is abnormally increased in FA cells. Reduction of these responses through MSH2 depletion also correlates with the restart of DNA replication. These findings have important clinical implications for BRCA-FA mutation carriers, as MSH2 inactivation might propel tumor formation or reduce the efficacy of platinum therapies used to treat BRCA-FA pathway-associated cancers.

## Results

### Loss of MSH2, but not MLH1 or NHEJ proteins, reduces the MMC sensitivity of cells deficient in FANCJ

**Figure 1:**
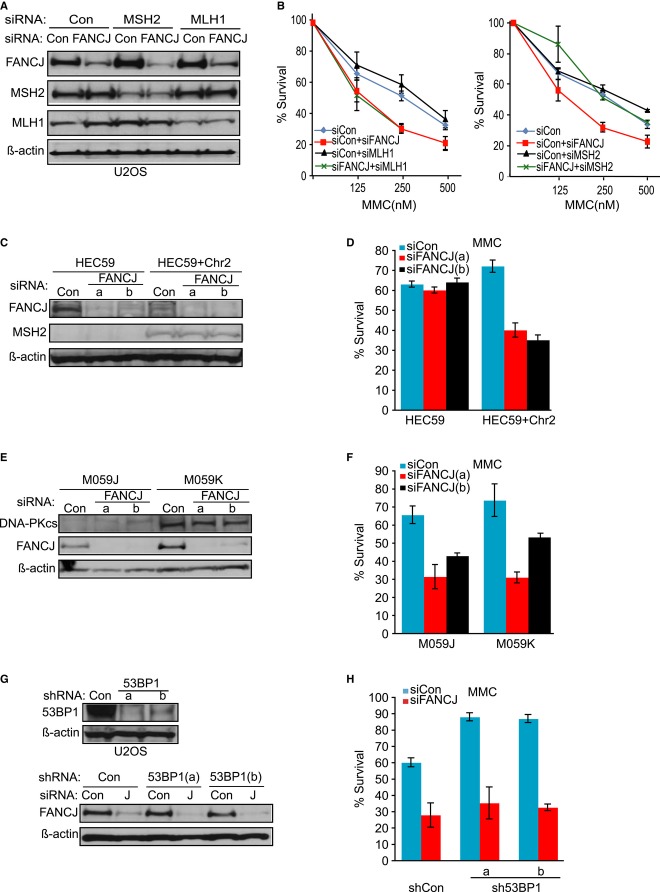
Sensitivity to mitomycin C (MMC) in FANCJ-deficient cells is suppressed by loss of MSH2, but not MLH1, DNA-PKcs, or 53BP1 Immunoblot analysis of FANCJ, MLH1, and MSH2 expressions in U2OS cells treated with indicated siRNAs. β-actin was used as a loading control.Graph shows the percentage of viable cells 5 days after indicated dose of MMC.Immunoblot analysis of FANCJ and MSH2 expressions in human MSH2-null (HEC59) and MSH2-proficeint (HEC59+Chr2) cell lines treated with indicated siRNA reagents to Con or FANCJ (a or b).Graph shows the percentage of viable cells 5 days after 500 nM MMC.Immunoblot analysis of FANCJ and DNA-PKcs in DNA-PKcs-deficient (M059J) and DNA-PKcs-proficient (M059K) cells treated with siRNA reagents to Con or FANCJ (a or b).Graph shows the percentage of viable cells 5 days after 250 nM MMC.Immunoblot analysis showing 53BP1 or FANCJ expression in U2OS cells stably expressing shRNA vectors to either control or 53BP1 (a or b) that were also transfected with siRNAs to Con or FANCJ.Graph shows the percentage of viable cells 5 days after 250 nM MMC. Data information: Where shown, error bars represent standard deviations from three independent experiments.

The diversity of MMR functions in ICL processing, including activation of the BRCA-FA pathway and converting ICLs into breaks (Fram *et al*, 1985; Nowosielska & Marinus, 2005; Zhang *et al*, 2007; Huang *et al*, 2011; Williams *et al*, 2011), suggests that pathway coordination is essential. To test the idea that MMR is toxic in the absence of coordination with the BRCA-FA pathway, we tested whether the ICL sensitivity of FANCJ-deficient cells is due to MMR factors. We used siRNA reagents to disrupt upstream and downstream MMR complexes, through MSH2 or MLH1 depletion, respectively. When siRNAs to FANCJ and MLH1 or MSH2 were used in combination or alone, knockdown was achieved (Fig1A). The reduction in FANCJ expression resulted in the expected sensitivity to MMC as compared to control (Fig1B). Furthermore, MMR depletion was sufficient to promote hyper-resistance to the DNA methylating agent methylnitrosourea (MNU; Brown *et al*, 1997; Supplementary Figure S1A) indicating the siRNAs decreased the function of these proteins. Strikingly, as compared to FANCJ depletion, FANCJ and MSH2 co-depletion enhanced MMC resistance, whereas FANCJ and MLH1 co-depletion did not (Fig1B and Supplementary Figure S1B). Substantiating these findings, a similar result was obtained in A549 cells with shRNA reagents targeting a distinct MSH2 site (Supplementary Figure S1C–E), indicating that these results were not likely to be cell type specific or the result of off-target effects.

To further validate these findings, we examined a pair of cell lines derived from a colon cancer patient, HEC59 (MSH2-deficient) and HEC59+chr2 (MSH2-proficient). HEC59 cells do not express MSH2 unless chromosome 2 is re-introduced (Fig1C; Umar *et al*, 1997). We found that treatment of HEC59+chr2 cells with either of two individual FANCJ siRNAs (a or b) resulted in sensitivity to MMC as compared to treatment with control siRNA (Fig1D). In contrast, MSH2-deficient HEC59 cells depleted of FANCJ exhibited the same levels of survival after MMC treatment comparable to controls. Altogether, these findings indicate that MSH2 contributes to the ICL sensitivity of FANCJ-deficient cells.

In light of recent studies in which elimination of the NHEJ factors, DNA-PKcs or 53BP1 reduced ICL sensitivity in BRCA-FA cells (Adamo *et al*, 2010; Bunting & Nussenzweig, 2010; Aly & Ganesan, 2011), we asked whether loss of these NHEJ factors also reduced ICL sensitivity in FANCJ-deficient cells. For these studies, we exploited the human M059K (DNA-PKcs proficient) and M059J (DNA-PKcs deficient) glioblastoma cell lines (Anderson *et al*, 2001). Treatment of the M059K or M059J cells with two different FANCJ siRNAs resulted in sensitivity to MMC (Fig1E and F). Likewise, treating the two stably 53BP1-depleted cell lines with FANCJ siRNA resulted in MMC sensitivity (Fig1G and H). Thus, eliminating or depleting DNA-PKcs or 53BP1 does not suppress MMC sensitivity associated with FANCJ deficiency, consistent with findings in *Caenorhabditis elegans* (Adamo *et al*, 2010).

### MSH2 depletion suppresses ICL sensitivity in cells lacking the FANCJ–MLH1 interaction

**Figure 2:**
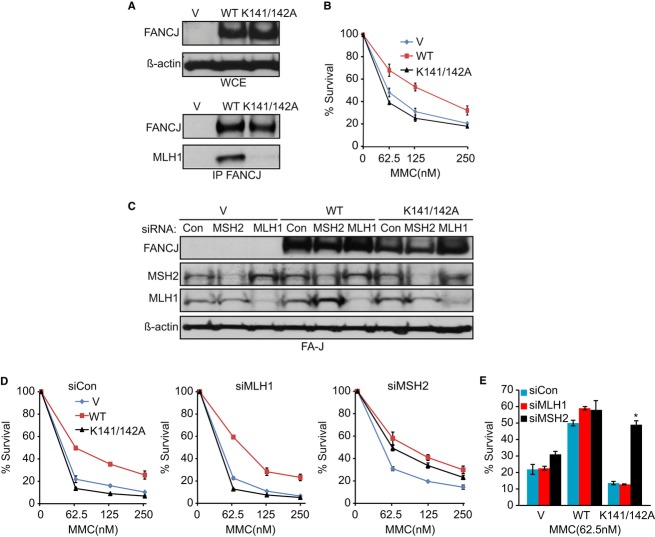
Mitomycin C (MMC)-induced sensitivity in cells lacking the FANCJ–MLH1 interaction is suppressed by MSH2 depletion A Immunoblot analysis of FANCJ and/or MLH1 in the indicated Fanconi anemia (FA)-J complemented cell lines from whole-cell extracts or following immunoprecipitation. B Graph shows the percentage of viable cells 5 days after increasing doses of MMC. C Immunoblot analysis of FANCJ, MSH2, and MLH1 expressions in the FA-J cell lines treated with the indicated siRNAs. D, E Graphs show the percentage of viable cells 5 days after increasing doses of MMC (D) or as compared at one dose (E). Data information: Where shown, error bars represent standard deviations from three independent experiments. The asterisk (*) represents a *P*-value < 0.01.

FANCJ binds directly to MLH1, and cells expressing a FANCJ mutant that cannot bind MLH1 are hypersensitive to MMC (Peng *et al*, 2007). Thus, we hypothesized that a function of the interaction was to inhibit the action of MSH2 at lesions induced by MMC. Immunoblot and co-immunoprecipitation experiments of FA-J-null (FA-J) patient cell lines confirmed that FANCJ^K141/142A^ was expressed similarly to FANCJ^WT^, but was defective in MLH1 binding (Fig2A). As expected, FA-J cells complemented with FANCJ^K141/142A^ remained sensitive to MMC, whereas FA-J cells complemented with wild-type FANCJ had enhanced resistance (Fig2B; Peng *et al*, 2007). Consistent with a rescue from MMC sensitivity, siRNA to MSH2 increased MMC resistance 3.6-fold in FA-J cells expressing the FANCJ^K141/142A^ mutant (Fig2C–E). In contrast, siRNA for MLH1 had no effect on MMC resistance in the FANCJ^K141/142A^ FA-J cell lines, indicating again that with respect to rescue from MMC sensitivity, MLH1 is distinct from MSH2.

In contrast to FA-J cells complemented with FANCJ^K141/142A^, MSH2 depletion had only a small effect on FA-J cells complemented with vector (Fig2D and E) and did not enhance MMC resistance in FA-J cells expressing a helicase inactivating mutant FANCJ^K52R^ (Supplementary Figure S2B). These findings suggest that MSH2 depletion rescues the repair defect in cells in which FANCJ cannot bind to MLH1, but does not rescue loss of a FANCJ function(s) connected to its helicase activity. Perhaps MSH2 depletion is able to rescue FANCJ-depleted cells because there is sufficient residual helicase activity (Fig1B and Supplementary Figure S1B).

### MSH2 depletion rescues aberrant checkpoint and DNA damage responses in cells lacking the FANCJ–MLH1 interaction

**Figure 3:**
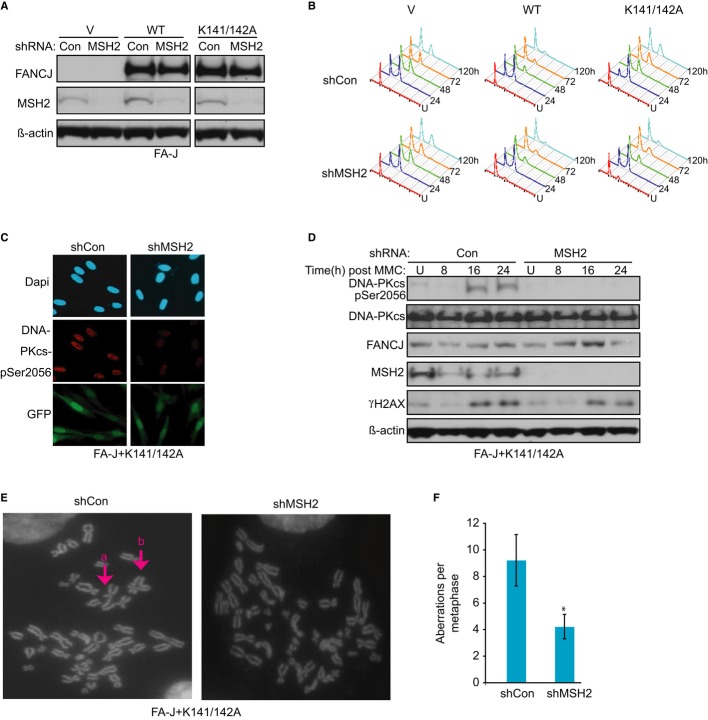
Aberrant DNA damage responses in cells lacking the FANCJ–MLH1 interaction are suppressed by MSH2 depletion Immunoblot analysis of FANCJ and MSH2 expressions in the Fanconi anemia (FA)-J cell lines expressing indicated shRNAs.Representative cell cycle profiles based on PI staining of DNA content for the indicated FA-J cell lines untreated (U) or at the indicated times following 0.25 μg/ml melphalan treatment.MSH2 depletion reduces DNA-PKcs Ser2056 phosphorylation after mitomycin C (MMC) treatment. Green fluorescent protein (GFP) expression indicates shRNA vector-infected FANCJ^K141/142A^ FA-J cells. Representative immunofluorescence images are shown.Immunoblot analysis with indicated antibodies.Genomic instability is suppressed by MSH2 depletion after 250 nM MMC for 16 h. Representative metaphases show examples of (a) broken and (b) quad-radial chromosomes that were suppressed by MSH2 depletion.Graph shows number of breaks and radials quantified from 50 metaphases. Data information: Where shown, error bars represent standard deviations from three independent experiments. The asterisk (*) represents a *P*-value < 0.01.

FA cells also have prolonged checkpoint responses and exacerbated DNA damage responses that are thought to contribute to the growth defects, ICL sensitivity, and bone marrow failure in FA patients (Ceccaldi *et al*, 2012; Kim & D'Andrea, 2012). The prolonged G2/M arrest in FA cells has been analyzed in response to ICL-inducing agents such as MMC or melphalan (Litman *et al*, 2005). We previously found that FA-J cells have a prolonged G2/M accumulation in response to melphalan that is corrected by introduction of FANCJ^WT^, but not the MLH1 binding mutant, FANCJ^K141/142A^ (Peng *et al*, 2007; Supplementary Figure S3). Moreover, as compared to vector, FANCJ^K141/142A^ complemented FA-J cells have a more pronounced G2/M accumulation (Peng *et al*, 2007; Supplementary Figure S3). Thus, we asked whether these cell cycle recovery defects were also attributable to MSH2. While MSH2 depletion had only a modest effect on FA-J cells with vector, and little or no effect on FANCJ^WT^, the prolonged G2/M accumulation was reduced in FA-J cells with FANCJ^K141/142A^ (Fig3A and B), suggesting that MSH2 contributes to the ICL-induced checkpoint defect in cells lacking the FANCJ–MLH1 interaction.

The exacerbated DNA damage response in FA cells includes hyper-phosphorylation of the NHEJ protein, DNA-PKcs (Adamo *et al*, 2010). To address whether MSH2 contributes to this DNA damage response, we generated FA-J cells with FANCJ^K141/142A^ and shRNA to MSH2 or control using vectors that also express green fluorescent protein (GFP). Cells positive for GFP and containing shRNA to MSH2 had markedly reduced MMC-induced DNA-PKcs phosphorylation relative to cells expressing shRNA control vector (Fig3C). Immunoblot analysis revealed a similar reduction in phosphorylation of DNA-PKcs and that MMC treatment induced a damage response evidenced by phosphorylated H2AX, γ-H2AX, albeit slightly less in the MSH2-depleted cells (Fig3D). These findings suggest that MSH2 mediates aberrant activation of NHEJ, perhaps by inducing DSBs in response to MMC.

Because NHEJ contributes to ICL-induced chromosomal aberrations in FA cells (D'Andrea & Grompe, 2003), we next tested whether MSH2 depletion limited the number or type of aberrations found in FA-J cells lacking the FANCJ–MLH1 interaction. Remarkably, we found fewer radial chromosomes and chromosomes with breaks induced by MMC treatment in the FANCJ^K141/142A^-complemented FA-J cells expressing shMSH2 than in cells expressing control shRNA (shCon; Fig3E). Cells expressing control shRNA had ˜9 chromosomal aberrations per mitotic spread as compared to ˜4 in cells expressing MSH2 shRNA (Fig3F). Collectively, these findings indicate that MSH2 contributes to the MMC sensitivity, prolonged G2/M accumulation, hyper-activation of DNA-PKcs, and radial chromosomes in FA-J cells lacking the FANCJ–MLH1 interaction.

**Figure 4:**
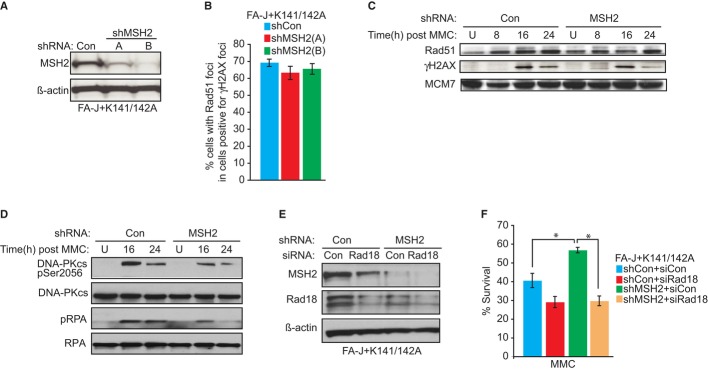
MSH2 depletion does not enhance RAD51 foci, but suppresses mitomycin C (MMC) sensitivity through a Rad18-dependent mechanism A Immunoblot analysis of MSH2 expression in the FANCJ^K141/142A^ Fanconi anemia (FA)-J cells treated with the indicated shRNAs. B Quantification of the percentage of γ-H2AX foci-positive cells that have also RAD51 foci after 250 nM MMC treatment for 16 h. C, D Immunoblot analysis with the indicated Abs of chromatin fractions from FANCJ^K141/142A^ FA-J cells stably expressing shRNA to Con or MSH2. E Immunoblot analysis of Rad18 and MSH2 expressions in FANCJ^K141/142A^ FA-J cells stably expressing shRNA to Con or MSH2 treated with indicated siRNAs. F Graph shows the percentage of viable cells 5 days after 125 nM MMC. Data information: Where shown, error bars represent standard deviations from three independent experiments. The asterisk (*) represents a *P*-value < 0.01.

MSH2 depletion could promote survival of MMC-treated cells by enhancing recombination-based repair. To address this possibility, FANCJ^K141/142A^-expressing FA-J cells positive for γ-H2AX foci were analyzed for co-staining Rad51 foci. At all time points examined, we found that cells positive for γ-H2AX foci have a similar percent of Rad51 foci whether they express shRNA control or MSH2 shRNA vectors (Fig4A and B, and Supplementary Figure S4A). Moreover, at several time point post-MMC, the chromatin bound Rad51 appeared similar and γ-H2AX was induced in both cell lines at 16 h post-MMC (Fig4C). While γ-H2AX was slightly reduced by 24 h post-MMC in MSH2-depleted FANCJ^K141/142A^-FA-J cells as compared to control FA-J cells, more striking was the reduction in phosphorylated RPA and DNA-PKcs (Fig4D and Supplementary Figure S4B and C). Thus, MSH2 does not appear to alter the accumulation of Rad51, perhaps at resected DNA sites prior to ICL excision (Long *et al*, 2011), but may contribute to ICL-induced break formation.

### MSH2 depletion reduces MMC sensitivity through a Rad18-dependent mechanism

The restart of stalled replication forks by TLS was shown to mediate the reduced cytotoxicity of cisplatin in MMR-deficient cells (Lin *et al*, 2006). TLS requires PCNA ubiquitination by the Rad18/Rad6 ubiquitin–ligase complex (Kannouche & Lehmann, 2004; Alt *et al*, 2007; Waters *et al*, 2009). Thus, we tested whether the gains in MMC resistance due to MSH2 depletion in the FANCJ^K141/142A^-complemented FA-J cells are dependent on Rad18. When siRNAs and shRNAs to Rad18 or MSH2 were used in combination or alone, knockdown was achieved (Fig4E). Compared with MSH2 depletion alone, MSH2 and Rad18 co-depletion reduced MMC resistance (Fig4F). Furthermore, gain in MMC resistance in MSH2-deficient HeLa cells was also dependent on Rad18 (Supplementary Figure S5A–D). Depleting Rad18 or the TLS polymerase REV1 also sensitized MCF7 or U20S cells co-deficient in FANCJ and MSH2 (Supplementary Figure S5E and F). These findings suggest that in a non-cell-type-specific manner, MSH2 depletion promotes MMC resistance through a Rad18-dependent mechanism that requires TLS polymerases such as REV1.

### MSH2 depletion restores replication restart after aphidicolin-induced arrest

**Figure 5:**
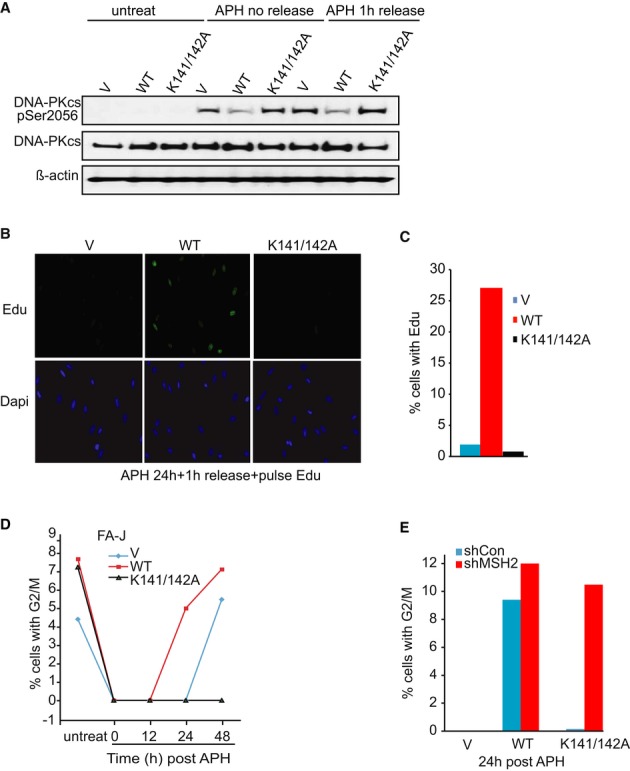
Fanconi anemia (FA)-J cells lacking the FANCJ–MLH1 interaction have a pronounced cell cycle progression defect that is suppressed by MSH2 depletion A Immunoblot analysis of phosphorylation of DNA-PKcs in the FA-J cell lines before or after aphidicolin (APH) treatment and release. B, C Immunofloresence representative figure (B) and quantification (C) after 18 h APH (3 μg/ml) treatment and 1 h release in the presence of EdU shows the FA-J cell lines differ in their ability to restore DNA synthesis. Over 300 cells per experiment were counted. D Graph shows the percentage of cells with 4N DNA content after indicated time following APH release. E MSH2 deletion suppresses the APH-induced cell cycle progression defect in FANCJ^K141/142A^ expressing FA-J cells. Graph shows the percent cells with 4N DNA content 24 h following APH release in shRNA-treated cell lines.

Processing of ICLs is complex and involves lesion processing, repair, and the restoration of replication fork progression. By simply arresting cells with the DNA polymerase inhibitor aphidicolin, we sought to gain clarity as to the underlying defect in cells lacking the FANCJ–MLH1 interaction. Moreover, it was previously shown that defects in S phase progression characterize FANCJ-deficient cells following release from aphidicolin (Greenberg *et al*, 2006; Kumaraswamy & Shiekhattar, 2007). Similar to treatment with MMC, treatment with aphidicolin correlated with an aberrant induction of DNA-PKcs phosphorylation in FA-J cells lacking FANCJ^WT^ (Fig5A and Supplementary Figure S6A). Correspondingly, FA-J cells lacking FANCJ^WT^ were severely defective in S phase progression following release from aphidicolin and did not incorporate Edu in a 1-h pulse (Fig5B and C). Notably, as compared to vector, FANCJ^K141/142A^-complemented FA-J cells had an even more severe defect and did not recover by 48 h, but rather required from 72 to 96 h to gain a 4N DNA population (Fig5D and data not shown), indicating that cells lacking the FANCJ–MLH1 interaction have a pronounced defect in resuming cell cycle progression. By analyzing the percent of G2/M cells following aphidicolin release, we found that MSH2 depletion effectively restored cell cycle progression to the FANCJ^K141/142A^-expressing FA-J cells (Fig5E) consistent with MSH2 interfering with the restart of stalled replication forks when cells lack the FANCJ–MLH1 interaction (see model in Supplementary Figure S7).

### MSH2 depletion reduces MMC sensitivity in human cells deficient in FANCJ, FANCD2, or BRCA1, but not FANCA

**Figure 6:**
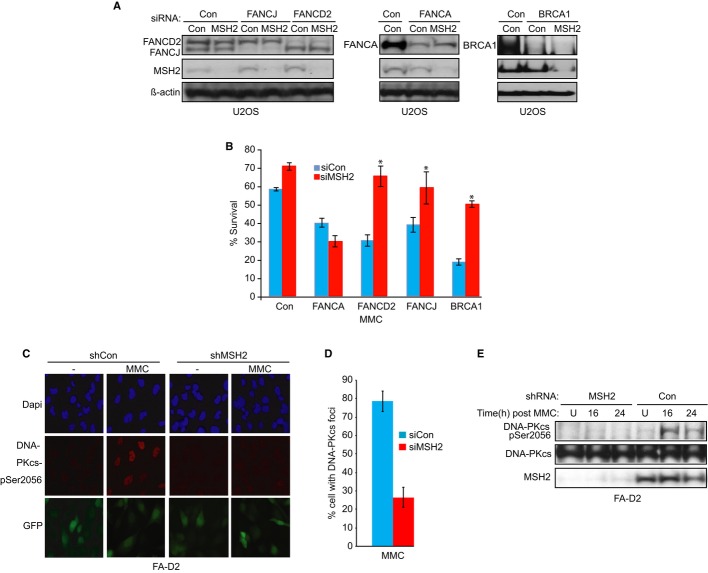
MSH2 depletion suppresses mitomycin C (MMC) sensitivity of cells deficient for FANCD2 or BRCA1, but not FANCA, and rescue correlates with reduced DNA-PKcs phosphorylation in FANCD2-deficient cells A Immunoblot analysis of FANCJ, FANCD2, BRCA1, FANCA, and MSH2 expressions in U2OS cells treated with indicated siRNA. B Graph shows the percentage of viable cells 5 days after 500 nM MMC. The asterisk (*) represents a *P*-value < 0.01. C, D Fanconi anemia (FA)-D2 (FANCD2-null) (PD20) patient cells stably expressing shRNA vectors with green fluorescent protein (GFP; control) or MSH2 were treated with 250 nM MMC for 16 h or left untreated. GFP expression indicates shRNA vector-infected cells. Representative immunofluorescence images (C) and quantification (D) of the percentage of cells with DNA-PKcs foci after 250 nM MMC treatment for 16 h. E Immunoblot analysis with the indicated Abs of chromatin fractionated FA-D2 cells stably expressing shRNA to Con or MSH2. Data information: Where shown, error bars represent standard deviations from three independent experiments.

To determine whether MSH2 underlies aberrant responses in other BRCA-FA cells, we tested whether MSH2 contributed to MMC sensitivity in cells lacking BRCA-FA proteins known to interact with MMR proteins, such as BRCA1 or the central FA pathway protein, FANCD2 (Wang *et al*, 2000; Williams *et al*, 2011). We also tested whether MSH2 contributed to MMC sensitivity in cells lacking the FA upstream core component, FANCA, which has not been shown to interact with MMR proteins. As expected, U2OS cells treated with siRNA reagents to FANCJ, FANCD2, FANCA, or BRCA1 were more sensitive to MMC than cells treated with control siRNA (Fig6A and B, blue bars). Similar to our results in FANCJ-deficient cells, BRCA1- or FANCD2-deficient U2OS cells treated with siRNAs to MSH2 have improved survival after MMC treatment as compared to cells treated with siRNA controls (Fig6B, red bars, Supplementary Figure S8A). Furthermore, the MMC sensitivity of BRCA1-depleted MCF7 cells was fully suppressed by MSH2 depletion (Supplementary Figure S8B and C). In contrast, the MMC sensitivity of FANCA-deficient U2OS or FA-A patient cells depleted of MSH2 was not suppressed (Fig6A and B, and Supplementary Figure S8D–G), suggesting FANCA functions in ICL processing in a manner distinct from FANCJ, BRCA1, or FANCD2.

DNA-PKcs is aberrantly phosphorylated in FANCD2-deficient cells in response to MMC (Adamo *et al*, 2010), similar to our findings in FANCJ mutant cells (Fig 3C and D). Thus, we asked whether MSH2 contributed to this aberrant response in FANCD2-deficient cells, as it does in FANCJ mutant cells. Indeed, FA-D2 patient cells stably expressing shRNA GFP-fusion vectors to MSH2 show markedly less MMC-induced DNA-PKcs phosphorylation than cells expressing shRNA control vector after MMC treatment (Fig6C and D). In addition, MSH2-depleted FA-D2 cells as compared to control FA-D2 cells had a reduction in the phosphorylation of DNA-PKcs (Fig6E), suggesting that MSH2 contributes to the abnormal DDR in FANCD2-deficient cells.

### Msh2 deletion reduces MMC sensitivity and the DNA damage response in Fancd2-null mouse cells

**Figure 7:**
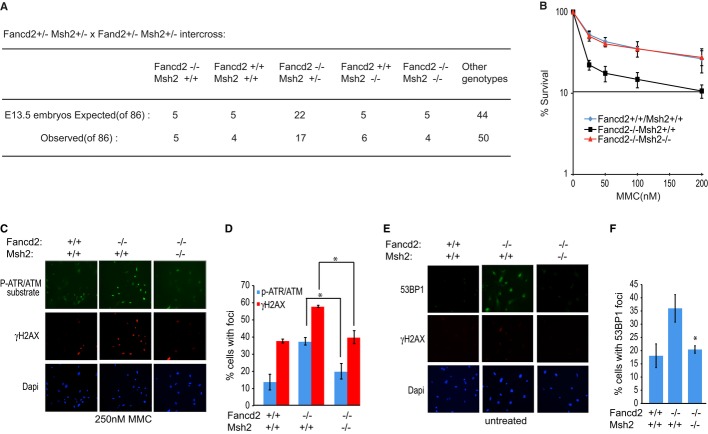
Sensitivity to mitomycin C (MMC) and the aberrant DNA damage response in Fancd2-null mouse cells are suppressed by Msh2 deletion A Chart shows embryos obtained from indicated cross. B Graph shows the percentage of viable primary mouse embryonic fibroblasts (MEFs) with the designated genotypes 5 days after MMC treatment. Three independent MEFs per genotype were analyzed. C, D Representative immunofluorescence images (C) and quantification (D) of cells with foci positive for the ATM/ATR substrate phosphorylation and γ-H2AX following 250 nM MMC treatment. E, F Representative immunofluorescence images (E) and quantification (F) of cells with 53BP1 foci and γ-H2AX in untreated MEFs. Data information: Where shown, error bars represent standard deviations from three independent experiments. The asterisk (*) represents a *P*-value < 0.01.

To substantiate these findings, we analyzed the impact of deleting *Msh2* in Fancd2-null mouse embryonic fibroblasts (MEFs). When mice heterozygous for *Fancd2* and *Msh2* were interbred and embryos were harvested between 13 and 14 days of gestation, we identified double-mutant embryos, which upon visual inspection resembled wild-type embryos. From 86 embryos, we obtained four double mutants (Fig7A). We found that compared with wild-type, the *fancd2^−/−^* MEFs were extremely sensitive to MMC, with only 22% of cells surviving after treatment with 25 nM MMC. We also found that resistance to MMC was enhanced in double-knockout MEFs to a level comparable with wild-type (Fig7B), indicating that ICL sensitivity is reduced by *Msh2* deletion as previously found for *Mlh1* deletion (van de Vrugt *et al*, 2009).

To further ascertain whether FA-like phenotypes found in Fancd2-null MEFs were suppressed by *msh2* deletion, we analyzed the DNA damage response. Compared with wild-type MEFs, MMC induced a greater damage response in *fancd2^−/−^* MEFs, as detected by an antibody to ATR/ATM-phosphorylated substrates and γ-H2AX, consistent with a previous report (Ceccaldi *et al*, 2012). These phosphorylation events were reduced in the *fancd2^−/−^msh2^−/−^* MEFs (Fig7C and D). Next, we analyzed 53BP1 foci, which mark unrepaired lesions remaining due to problems during replication (Lukas *et al*, 2011). As reported in FANCD2-deficient human cells (Ceccaldi *et al*, 2012) and FANCM-deficient cells (Blackford *et al*, 2012), we found more 53BP1 foci in the *fancd2^−/−^* MEFs than in wild-type MEFs. And here too, we detected fewer 53BP1 foci in the *fancd2^−/−^msh2^−/−^* MEFs (Fig7E and F). Together, these findings suggest that Msh2 contributes to ICL sensitivity and the heightened DNA damage response in Fancd2-null cells.

## Discussion

The BRCA-FA and MMR pathways intersect through several protein interactions. FANCD2-FAN1, BRCA1, and FANCJ interact with MLH1, and SLX4/FANCP interacts with MSH2 (Wang *et al*, 2000; Svendsen *et al*, 2009; Kratz *et al*, 2010; Liu *et al*, 2010; O'Donnell & Durocher, 2010; Shereda *et al*, 2010; Smogorzewska *et al*, 2010; Yoshikiyo *et al*, 2010). Moreover, the MMR pathway activates the BRCA-FA pathway, inducing FANCD2 monoubiquitination and localization of FANCJ to sites of DNA crosslinks (Huang *et al*, 2011; Williams *et al*, 2011; Suhasini *et al*, 2013; Guillemette *et al*, 2014). In this study, we provide further evidence that crosstalk between BRCA-FA and MMR pathways is critical for coordinating the DNA damage response. In particular, we find that lack of coordination between these pathways, as in BRCA-FA cells, makes MSH2 toxic. Consistent with this point, ICL sensitivity is suppressed by MSH2 depletion in human cells deficient in the FANCJ–MLH1 interaction, FANCJ, or other BRCA-FA proteins, such as BRCA1 or FANCD2 that interact with MMR proteins (Figs1B, 2D and E). Also, eliminating the MMR pathway in Fancd2-deficient mouse cells suppresses ICL sensitivity (Fig7B; van de Vrugt *et al*, 2009), whereas loss of NHEJ does not (Houghtaling *et al*, 2005; Bunting *et al*, 2012). Interestingly, MSH2 depletion fails to rescue FANCA-deficient cells (Fig6B and Supplementary Figure S8D–G). Thus, in a distinct set of BRCA-FA pathway deficient cells, loss of MMR function could synergize and contribute to tumors in FA patients.

Our data indicate that in the absence of BRCA-FA and MMR pathway coordination, MSH2 exacerbates the DNA damage response that characterizes FA cells. In particular, FA cells have a dysregulated NHEJ pathway in which DNA-PKcs is hyper-phosphorylated in response to DNA damage (Adamo *et al*, 2010). Aberrant DNA damage responses also include hyper-induction of ATR/ATM signaling pathways (Lukas *et al*, 2011; Ceccaldi *et al*, 2012), which were apparent in the Fancd2-null MEFs following MMC (Fig7C and D). Even in unperturbed Fancd2-null MEFs, 53BP1 foci are enhanced, suggesting replication stress (Fig7E and F; Lukas *et al*, 2011). Significantly, these aberrant DNA damage responses are suppressed by MSH2 loss (Fig7C–F). Phosphorylation of DNA-PKcs is associated with its binding to DNA double-strand breaks (Meek *et al*, 2004). Therefore, DSB induction is likely a consequence of MSH2 activity in FA cells treated with MMC. In agreement, inhibiting MMR reduces the number of DSBs at stalled forks and promotes ICL resistance through enhanced recombination and TLS bypass pathways (Brown *et al*, 1997; Durant *et al*, 1999; Moreland *et al*, 1999; Wu *et al*, 2004; Lin *et al*, 2006). Likewise, we found that bypass pathways are essential for the mechanism of ICL resistance in MSH2-depleted cells (Fig4F and Supplementary Figure S5B–G). Thus, we propose that eliminating MSH2 and its associated DNA damage response enhances ICL resistance through bypass pathways.

Our results also indicate that MSH2 depletion does not dramatically improve Rad51-based repair. We did not find evidence that recombination was heightened, as MMC-induced Rad51 foci and chromatin loading did not change upon MSH2 deletion (Fig4B and C). In support of MSH2 blocking a restart step in ICL processing, when FANCJ mutant cells are arrested by aphidicolin, we also find induction of DNA-PKcs phosphorylation and defects in the restart of DNA replication (Fig5A–C). Upon MSH2 depletion, however, mutant FA-J cells progress to G2/M (Fig5E). Interestingly, MSH2 deficiency abolishes the anticancer and pro-aging activity of short telomeres by reversing proliferative defects, but not by enhancing recombination (Martinez *et al*, 2009). Thus, we suggest that in cells deficient for FANCJ, BRCA1, or FANCD2, MSH2 loss improves the ability of stalled forks to restart, but does not increase DNA repair.

The finding that MSH2-, but not MLH1-depletion rescues cells deficient for FANCJ or the FANCJ–MLH1 interaction (Figs1B and 2D) suggests that FANCJ functions with MLH1 to prevent corruption/blockage of ICL repair by MSH2. Conceivably, FANCJ helicase and/or translocase activities could displace MSH2 heterodimers or unwind DNA structures that are substrates for MSH2. Of note, MSH2 binds branched DNA structures associated with replication forks (Alani *et al*, 1997; Kolodner & Marsischky, 1999), including G4 DNA structures that are substrates for FANCJ unwinding (Larson *et al*, 2005; Wu *et al*, 2009; Sarkies *et al*, 2012). Thus, to temper an MSH2 DNA damage response, FANCJ could restrict MSH2 heterodimers in several ways. First, to promote ICL repair, FANCJ might remove MSH2 bound to ICLs. Second, to promote error-prone recombination or TLS extension reactions that are required for ICL processing (Sharma & Canman, 2012), FANCJ might remove MSH2 that is bound to DNA mismatches. Finally, to restart replication following ICL processing, FANCJ might similarly prevent MSH2 binding to secondary structures that form at arrested forks that are barriers to replication. Unwinding DNA substrates and/or displacing MSH2 could explain the role of FANCJ in coupling replication past fork barriers (Schwab *et al*, 2013). The ability of FANCJ to dismantle MSH2 could underlie the importance of the FANCJ–MLH1 interaction for ICL repair as MLH1 links FANCJ to the MSH2 heterodimer (Peng *et al*, 2007). We suspect that the relevant heterodimer displaced is MSH2–MSH6. This conclusion is not based on MSH6 depletion studies because MSH6 siRNAs also reduced MSH2 expression (Supplementary Figure S9), and thus, the experiment would be uninformative. However, MSH3 depletion did not alter MSH2 or MSH6 levels, and we found that MMC resistance was not enhanced (Supplementary Figure S9).

Aside from functioning with MLH1 to limit MSH2, our data suggest that FANCJ has a separate helicase function that is also important for ICL repair. Most notably, MSH2 depletion has little or no effect on FANCJ-null cells or cells that lack FANCJ helicase activity (Supplementary Figure S2B). This further supports the hypothesis that FANCJ functions with MLH1 to remove MSH2 from a DNA lesion, perhaps with its translocase activity, and also unwinds DNA barriers with its helicase activity. As compared to FANCJ-null cells, cells lacking the FANCJ–MLH1 interaction have a more severe ICL processing defect (Peng *et al*, 2007; Fig3B) and a more severe replication restart defect following arrest by aphidicolin treatment (Fig5D), suggesting that stalled replication forks are differentially processed (see model in Supplementary Figure S7). We speculate that stalled replication forks remain intact in FANCJ-null cells, a point supported by studies in DT40 cells (Schwab *et al*, 2013). Restart at these forks likely involves loss of genomic integrity, consistent with loss of G4 structures in FANCJ-null cells (Cheung *et al*, 2002; London *et al*, 2008; Wu *et al*, 2008; Sarkies *et al*, 2012). Replication barriers could be cleaved by opportunistic nucleases that gain access to stalled forks, as found in BRCA-FA cells (Schlacher *et al*, 2011, 2012). Instead, in cells lacking the FANCJ–MLH1 interaction, replication barriers could be insurmountable. FANCJ could physically block nucleases and MSH2 could in turn block FANCJ. Thus, restart via nucleases and break induction fails and forks collapse. This model suggests that in FANCJ^K141/142A^ mutant cells, MSH2 depletion, but not MLH1, will ‘unlock’ the secondary structure and enable FANCJ to unwind the replication barrier and restart replication. In FANCJ-null cells, MSH2 depletion has little effect because without FANCJ helicase activity, the replication barrier remains and restart will be largely dependent on nucleases. In sum, MSH2 depletion may rescue cells that have FANCJ helicase activity (FANCJ^K141/142A^ FA-J), but not cells without FANCJ helicase activity (vector or FANCJ^K52R^ FA-J cells). Rescue may be achieved in FANCJ siRNA-depleted cells because sufficient residual FANCJ supports its helicase function.

FANCD2-deficient cells are effectively rescued by loss of MSH2 (Figs6 and 7) or by loss of MLH1 when p53 is also inactivated (van de Vrugt *et al*, 2009). While MLH1 loss alone did not rescue cells deficient for FANCJ or the FANCJ–MLH1 interaction, we did not address if co-depletion of p53 would. Any differences could reflect the fact that some BRCA-FA proteins function with MMR complexes to initiate the DNA damage response aside from a role in regulating MMR. Indeed, FANCD2 forms complexes with MMR proteins at several nodes. FANCD2 binds MLH1 (Huang *et al*, 2011; Williams *et al*, 2011), and following DNA damage, the monoubiquitinated FANCD2 is found in a complex containing the MLH1-associated endonuclease FAN1 (Kratz *et al*, 2010; Smogorzewska *et al*, 2010). To process ICLs, FAN1 is predicted to function with other endonucleases, such as SLX4, that associate with MSH2 (Kim & D'Andrea, 2012). Thus, FANCD2 and associated partners could serve to link upstream and downstream MMR complexes. In FANCJ-deficient cells, in which FANCD2 undergoes a normal DNA damage-induced monoubiquitination (Litman *et al*, 2005), MLH1 loss could be insufficient to disarm an MSH2–SLX4–FANCD2–FAN1-dependent DNA damage response.

Our study indicates that MSH2 depletion rescues a subset of BRCA-FA cells, FANCJ-, BRCA1-, and FANCD2-deficient, but not FANCA-deficient cells. Notably, MSH2 loss also does not restore ICL resistance to FANCM-null DT40 cells (Huang *et al*, 2011). This distinction could reflect that FANCJ, BRCA1, and FANCD2 function at least in part through their MMR protein interactions, whereas to our knowledge, FANCA and FANCM do not interact with MMR proteins. BRCA1 and FANCD2, similar to FANCJ, could restart replication through MMR protein interactions that serve to dismantle MSH2. By contrast, FANCA and FANCM could contribute to replication restart by engaging bypass pathways. Indeed, FANCM is required for template switch mechanisms (Whitby, 2010; Blackford *et al*, 2012). Likewise, FANCA through complex formation with REV1 activates bypass pathways (Kim *et al*, 2012; Fig6F). A fundamental role in bypass pathways could explain why MSH2 depletion fails to rescue.

Taken together, these findings are relevant for understanding FA disease and progression to cancer. FA patient hematopoietic stem and progenitor cells have a hyperactivated DNA damage response that is dampened in transformed FA cells (Ceccaldi *et al*, 2012). Determining whether this abnormal DNA damage response and/or the progression to bone marrow failure in FA patients are generated by MMR factors that promote a barrier to tumorigenesis will be an important future research direction. If so, loss of MMR could predict the onset of tumorigenesis in patients. Moreover, it will be important to identify whether MMR inactivation or suppression will be useful for therapy to retard the progression to bone marrow failure.

## Materials and Methods

### Cell culture

MCF7, HeLa, A549, and U2OS cells were grown in DMEM supplemented with 10% fetal bovine serum and penicillin/streptomycin (100 U/ml each). M059J and M059K cells were cultured in DMEM/F12 (1:1) supplemented with 10% fetal bovine serum and antibiotics. Human endometrial HEC59 and HEC59+chr2 cell lines, FA-J (EUFA30-F), FA-D2 (PD20), and FA-A (PD6914) cell lines were cultured in DMEM supplemented with 15% fetal bovine serum. FA-J cells were infected with the POZ retroviral vectors as described in Nakatani & Ogryzko (2003). Generation of FANCJ^WT^, FANCJ^K52R^, FANCJ^K141/142A^, and pOZ vectors and stable FA-J cell lines was described (Peng *et al*, 2007). Stable shRNA cells were selected with puromycin.

### siRNA and shRNA

siRNA transfections were carried out with Lipofectamine RNAiMax (Invitrogen, Carlsbad, CA, USA) according to the manufacturer's instructions. Analyses were performed 48–72 h after siRNA transfection. siRNA reagents for MSH2 (siRNA MSH2 pool, target sequence of (a) GAAGAGACCUUAACUAUGC, or (b) GGAGGUAAAUCAACAUAUA), MLH1 (siRNA MLH1 smartpool), Rad18 (siRNA Rad18 smartpool), FANCD2 (siRNA FANCD2 smartpool), BRCA1 (siRNA BRCA1 smartpool), FANCA [siRNA FANCA smartpool or two distinct siRNA, target sequence of (a) AGAGGAAGAUGUUCACUUAUU, or (b) GGACAUCACUGCCCACUUCUU)], and luciferase (Luc) were obtained from Dharmacon (Lafayette, CO, USA). The FANCJ siRNA reagents were described previously (Litman *et al*, 2005). U2OS, FA-D2, or FA-J stable cells were infected with pGIPZ vectors expressing GFP and containing shRNAs against non-silencing control, MSH2 (a) (mature antisense sequence, CATGTAATAGAGTGTGCTA) or MSH2 (b) (ATTACTTCAGCTTTTAGCT), 53BP1(a) (mature antisense sequence, AGCAGCAACCCAGACTATA), or 53BP1 (b) (AGAAGTAGAAAGAAAAGTA), MSH6(a) (mature antisense sequence, TTCAACTCGTATTCTTCTGGC) or MSH6(b) (mature antisense sequence, TTTCAACTCGTATTCTTCTGG), MSH3(a) (mature antisense sequence, ATGACCTTATTCCTTCTGTGC) or MSH3(b) (mature antisense sequence, TTCCCTTAATTTAAGGAGTGG). shRNAs were obtained from the UMMS shRNA core facility.

### Cell growth and cell cycle analysis

Cells were seeded into six-well plates, incubated overnight, and left untreated or treated with MMC (Sigma, St Louis, MO, USA) for 1 h and CPT for 5 h (Invitrogen). Cells treated with MNU (1 h, serum free) were first pre-treated with 20 μM O6-benzylguanine (O6-BZG) to block methylguanine methyltransferase (MGMT). In addition, O6-BZG was also included during and after treatment. Cells were counted after 5 days using a hemocytometer and compared with untreated controls, and cell survival was analyzed as before (Xie *et al*, 2010). FA-J stable cell lines were either mock-treated or treated with 0.25 μg/ml of melphalan (Sigma) or 18 h with aphidocolin (3 μg/ml; Sigma) at which time fresh media was introduced. Cells were collected at various times and fixed with 90% methanol in PBS overnight and then incubated 10 min with PBS containing 30 μg/ml DNase-free RNase A and 50 μg/ml propidium iodide. 1 × 10^4^ cells were analyzed using a FACSCalibur instrument (Becton-Dickinson, San Jose, CA, USA). Aggregates were gated out, and the percentage of cells in G2/M was calculated using ModFit software. Errors represent standard deviation of the mean. Statistical analysis was performed using Student's two-tailed, unpaired *t*-test.

### Western blot and antibodies

Cells were harvested, lysed, and processed for immunoblot analysis as described previously using an NETN lysis buffer (20 mM Tris, 150 mM NaCl, 1 mM EDTA, and 0.5% NP-40) containing 10 mM NaF and 1 mM NaVO_3_ (Litman *et al*, 2005). Antibodies for Western blot analysis included FANCJ (E67), BRCA1 (ms110) (Cantor *et al*, 2004), MLH1 (Santa Cruz), MSH2 (Calbiochem), 53BP1 (Novus Biologicals), FANCD2 (FARF), FANCA (FARF), DNA-PKcs Ser2056 (Abcam), phospho RPA32 (S4/S8) (Bethyl), RPA32 (Bethyl), DNA-PKcs (Biolegend), MCM-7 (Abcam), γ-H2AX (Millipore), Rad18 (Abcam), Rad51 (Santa Cruz), MSH6 (BD Bioscience), MSH3 (BD Bioscience), and β-actin (Sigma). Chromatin preparations were carried out with NE-PER Nuclear and Cytoplasmic Extraction Reagents kit (Thermo Scientific) according to the manufacturer's instructions. The ratio of phospho-protein to total protein was measured and quantified using ImageJ software.

### Immnuofluorescence

Immunofluorescene was performed as described previously (Cantor *et al*, 2001). Cells grown on cover slips were either untreated or treated with MMC (250 nM) for 16 h. Then, cells were fixed and permeabilized. After incubation with primary antibodies against phospho DNA-PKcs (Abcam; 1:200), γ-H2AX (Upstate 1:100), Rad51 (Abcam; 1:1,000), 53BP1 (Novus Biologicals; 1:100), or P-ATR/ATM Substrate (Cell Signaling; 1:100), cells were washed and then incubated with secondary antibody. After washing, cover slips were mounted onto glass slides using Vectashield mounting medium containing DAPI (Vector Laboratories). Cells with > 10 foci per cell were scored as positive. For Edu labeling, cells were left untreated or treated with aphidocolin for 18 h and released at indicated times. Edu labeling was carried out with Click-iT Edu imaging kit (Invitrogen) according to the manufacturer's instructions.

### Metaphase spread preparation

Cells were left untreated or treated with MMC. Then, cells were incubated in media containing 100 ng/ml Colcemid for 1.5 h. After incubation, cells were harvested by trypsinization, lysed in 75 mM KCl, and fixed with fixative solution (75% methanol, 25% acetic acid). Fixed cells were dropped onto slides at 55°C, allowed to dry, and stained with Giemsa. Chromosome abnormalities were scored based on standard guidelines (Levitt *et al*, 2007).

### Generation of mice and PCR genotyping

Msh2-deficient mice (Reitmair *et al*, 1996) were obtained from T. Mak, University of Toronto, Toronto, Canada. Fancd2-deficient mice (Houghtaling *et al*, 2003) were obtained from M. Grompe, Oregon Health and Sciences University, Portland, OR, USA. The mouse strains were backcrossed to C57BL/6 for at least eight generations. Mice were maintained as heterozygotes, and double heterozygotes (*Msh2^+/−^Fancd2^+/−^)* were bred to obtain embryos of all six genotypes studied here. Mice were housed in the same room of the IACUC-approved SPF facility at University of Massachusetts Medical School and were bred and used under guidelines formulated by the University of Massachusetts Animal Care and Use Committee. As described in Reitmair *et al*, 1996 and Houghtaling *et al*, 2003, 50 ng of gDNA was prepared from embryo's head or mom's tail and used as a template in PCR to genotype mice. For *Fancd2*, forward primer MG968 (5′-TCAGCCTCACATGGAGTTTAACG-3′) and two reverse primers MG1280 (5′-GCTACACAGCATTGCCCATAAAG-3′) and MG1008 (5′-CAGGGATGAAAGGGTCTTACGC-3′) were used to amplify a wild-type band of 303 bp or a mutant band of 459 bp. The reaction conditions were 95°C for 2 min; 36 cycles of 94°C for 25 s, 58°C for 25 s, and 72°C for 35 s; and a final extension at 72°C for 2 min. For *Msh2*, forward primer *Msh2* COM (5′-AAAGTGCACGTCATTTGGA-3′) and two reverse primers *Msh2* WT (5′-GCTCACTTAGACGCCATTGT-3′) and *Msh2* MT (5′-GCCTTCTTGACGAGTTCTTC-3′) were used to amplify a wild-type band of 174 bp or a mutant band of 460 bp. The reaction conditions were 95°C for 2 min; 36 cycles of 94°C for 30 s, 62°C for 30 s, and 72°C for 30 s; and a final extension at 72°C for 7 min.
