# Supplementary material for: Crosstalk between BRCA-Fanconi anemia and mismatch repair pathways prevents MSH2-dependent aberrant DNA damage responses
Source: EMBO J. 2014 Jun 26;33(15):1698–712. doi: 10.15252/embj.201387530 (PMC4194102; doi:10.15252/embj.201387530)
Supplement: Supplementary file 1 [file embj0033-1698-sd1.pdf]

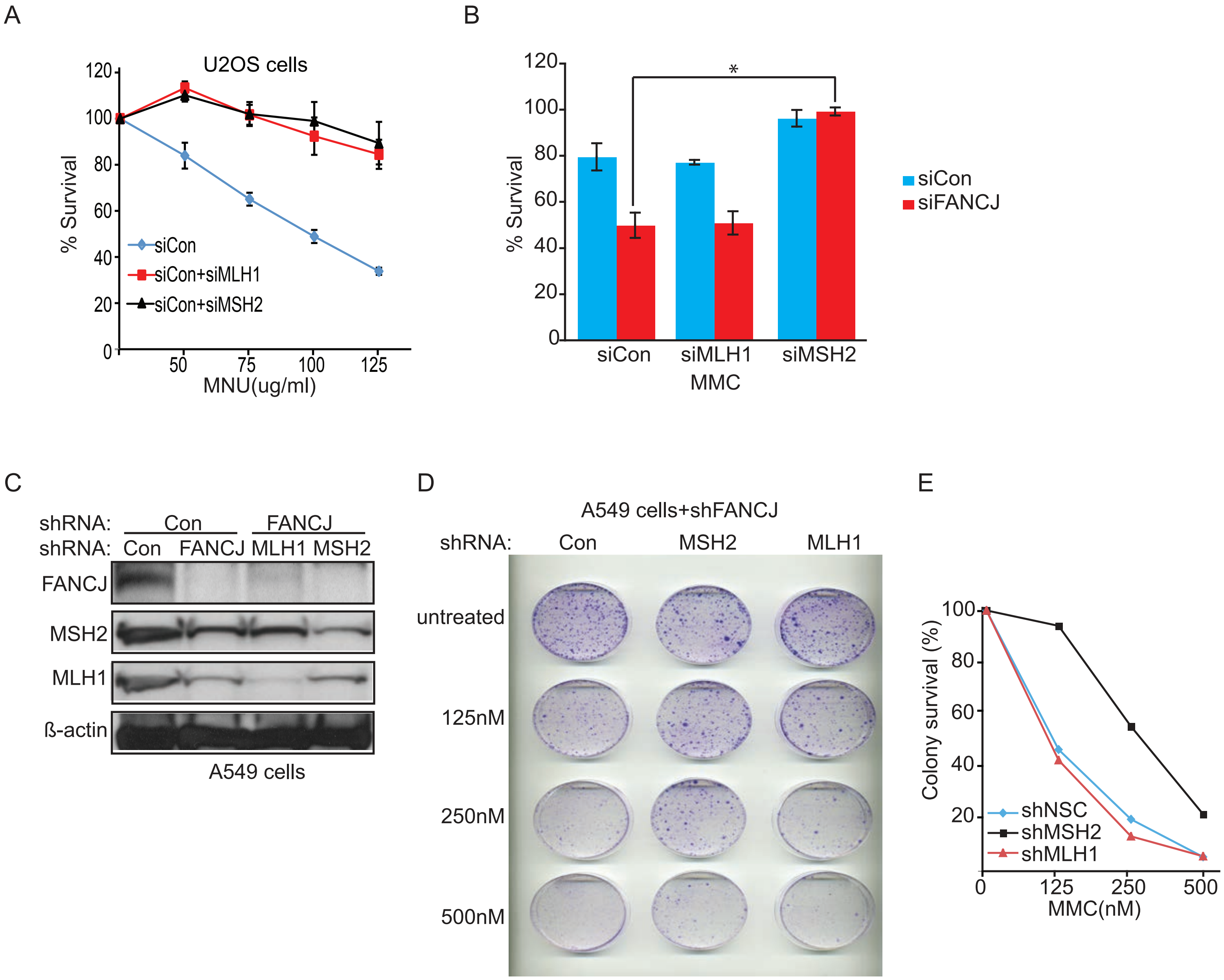

Supplementary Figure S1. **MSH2, but not MLH1 depletion suppresses the MMC sensitivity of FANCJ-deficient cells**. (A) Graph shows the percent-age survival 5 days after MNU or (B) MMC treatment in the U2OS cells transfected with the indicated siRNAs. (C) Immunoblot analysis of FANCJ, MSH2, and MLH1 expression in A549 cells treated with indicated shRNAs. (D) Colony survival assay shows enhanced resistance to MMC when MSH2 is depleted in FANCJ-deficient A549 cells as graphed in (E). Where shown, error bars represent standard deviations from three independent experi-ments. The asterisk (*) represents a p-value < 0.01.
