# Supplementary material for: Crosstalk between BRCA-Fanconi anemia and mismatch repair pathways prevents MSH2-dependent aberrant DNA damage responses
Source: EMBO J. 2014 Jun 26;33(15):1698–712. doi: 10.15252/embj.201387530 (PMC4194102; doi:10.15252/embj.201387530)
Supplement: Supplementary file 2 [file embj0033-1698-sd2.pdf]

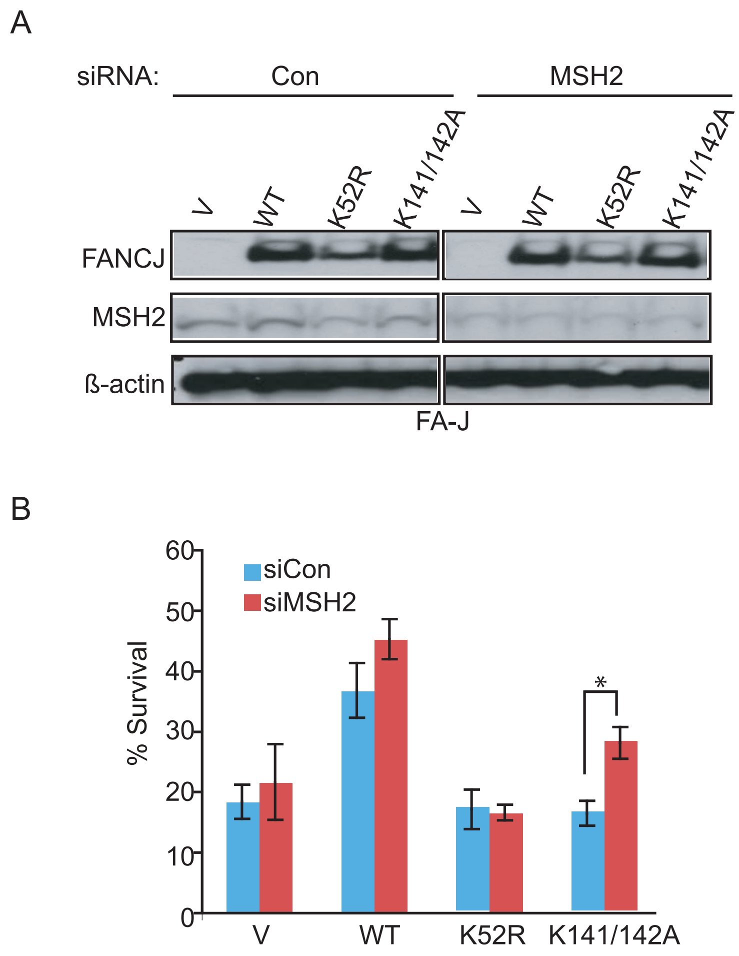

Supplementary Figure S2. MSH2 does not suppress MMC sensitivity in FA-J cells with inactive helicase FANCJ. (A) Immunoblot analysis of FANCJ and MSH2 expressionin the FA-J stable cell lines treated with indicated siRNAs.(B) Graph shows the percentage of viable cells 5days after MMC. Where shown, error bars represent standard deviations from three independent experiments. The asterisk (*) represents a p-value < 0.01.
