# Supplementary material for: Crosstalk between BRCA-Fanconi anemia and mismatch repair pathways prevents MSH2-dependent aberrant DNA damage responses
Source: EMBO J. 2014 Jun 26;33(15):1698–712. doi: 10.15252/embj.201387530 (PMC4194102; doi:10.15252/embj.201387530)
Supplement: Supplementary file 3 [file embj0033-1698-sd3.pdf]

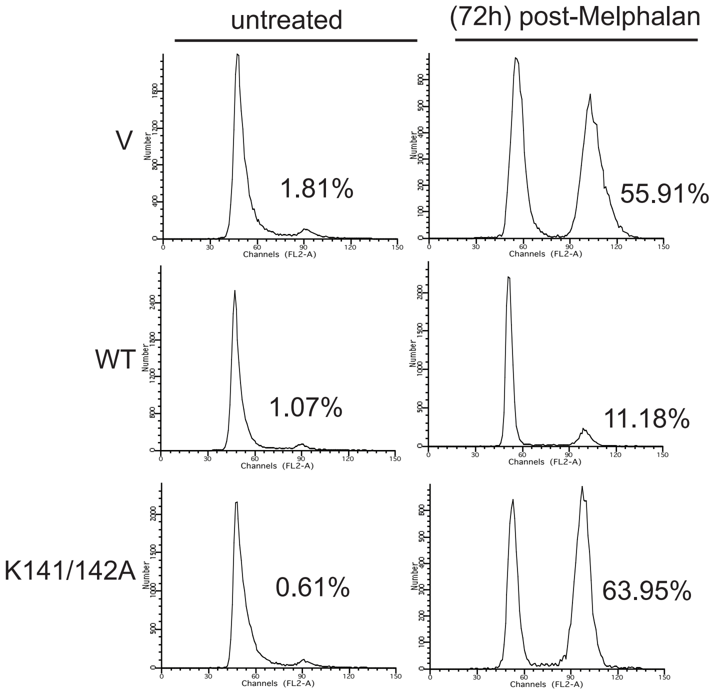

Supplementary Figure S3. FA-J cells complemented with FANCJK141/142A have an abnormal G2/M accumulation. The G2/M checkpoint in response to melphalan is shown in the distinct FA-J cell lines as the percent cells with 4N DNA content.
