# Supplementary material for: Crosstalk between BRCA-Fanconi anemia and mismatch repair pathways prevents MSH2-dependent aberrant DNA damage responses
Source: EMBO J. 2014 Jun 26;33(15):1698–712. doi: 10.15252/embj.201387530 (PMC4194102; doi:10.15252/embj.201387530)
Supplement: Supplementary file 4 [file embj0033-1698-sd4.pdf]

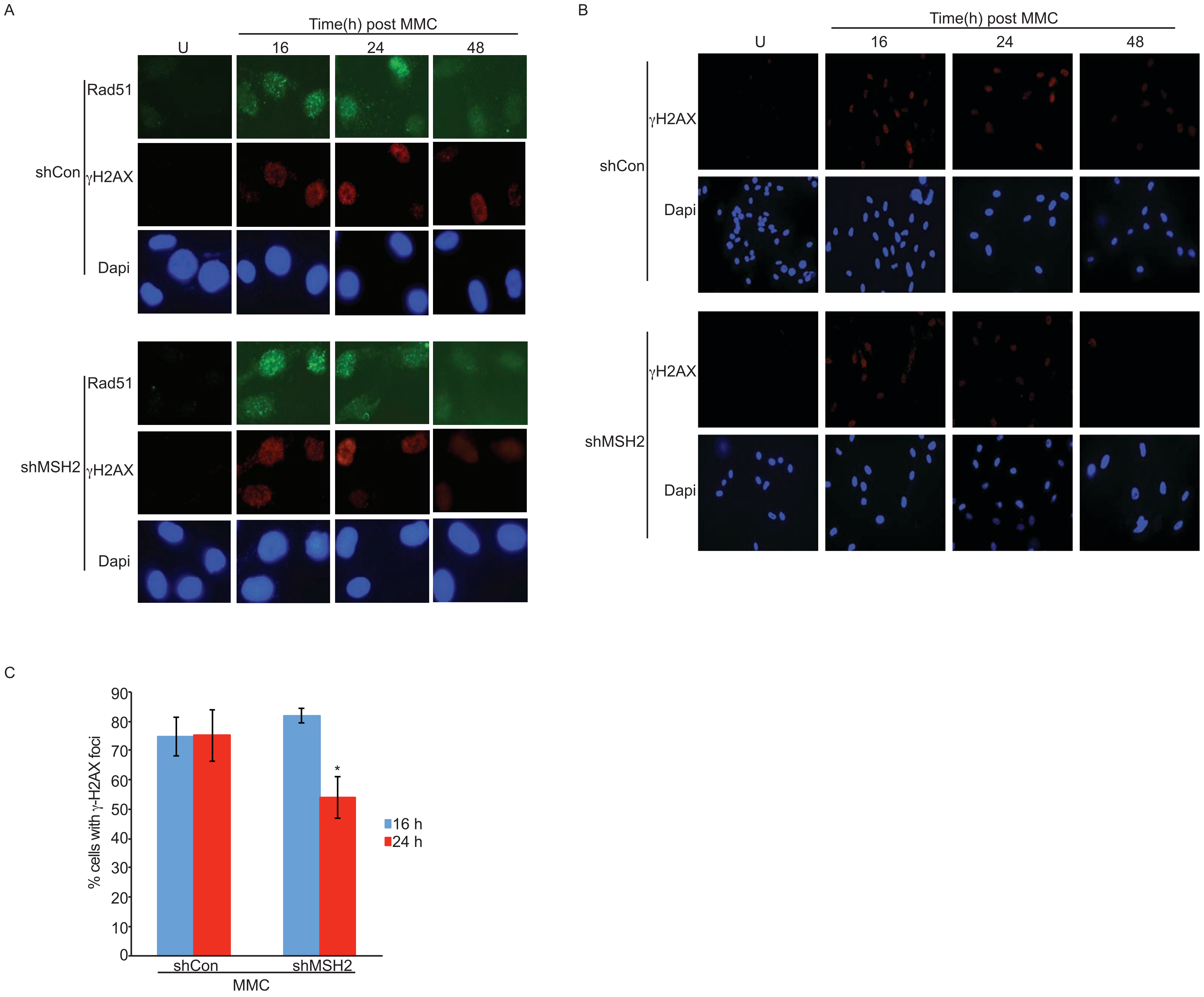

Supplementary Figure S4. MSH2 depletion does not enhance RAD51 foci, but modestly suppresses MMC induced γ-H2AX. Representative immunofluorescence images of FANCJK141/142A FA-J cells stably expressing shRNA vectors to control or MSH2 at the indicated time post-250nM MMC or left untreated that were either (A) selected based on positive γ-H2AX stain, (B) randomly selected and (C) quantitated. The asterisk (*) represents a p-value < 0.01.
