# Supplementary material for: Crosstalk between BRCA-Fanconi anemia and mismatch repair pathways prevents MSH2-dependent aberrant DNA damage responses
Source: EMBO J. 2014 Jun 26;33(15):1698–712. doi: 10.15252/embj.201387530 (PMC4194102; doi:10.15252/embj.201387530)
Supplement: Supplementary file 5 [file embj0033-1698-sd5.pdf]

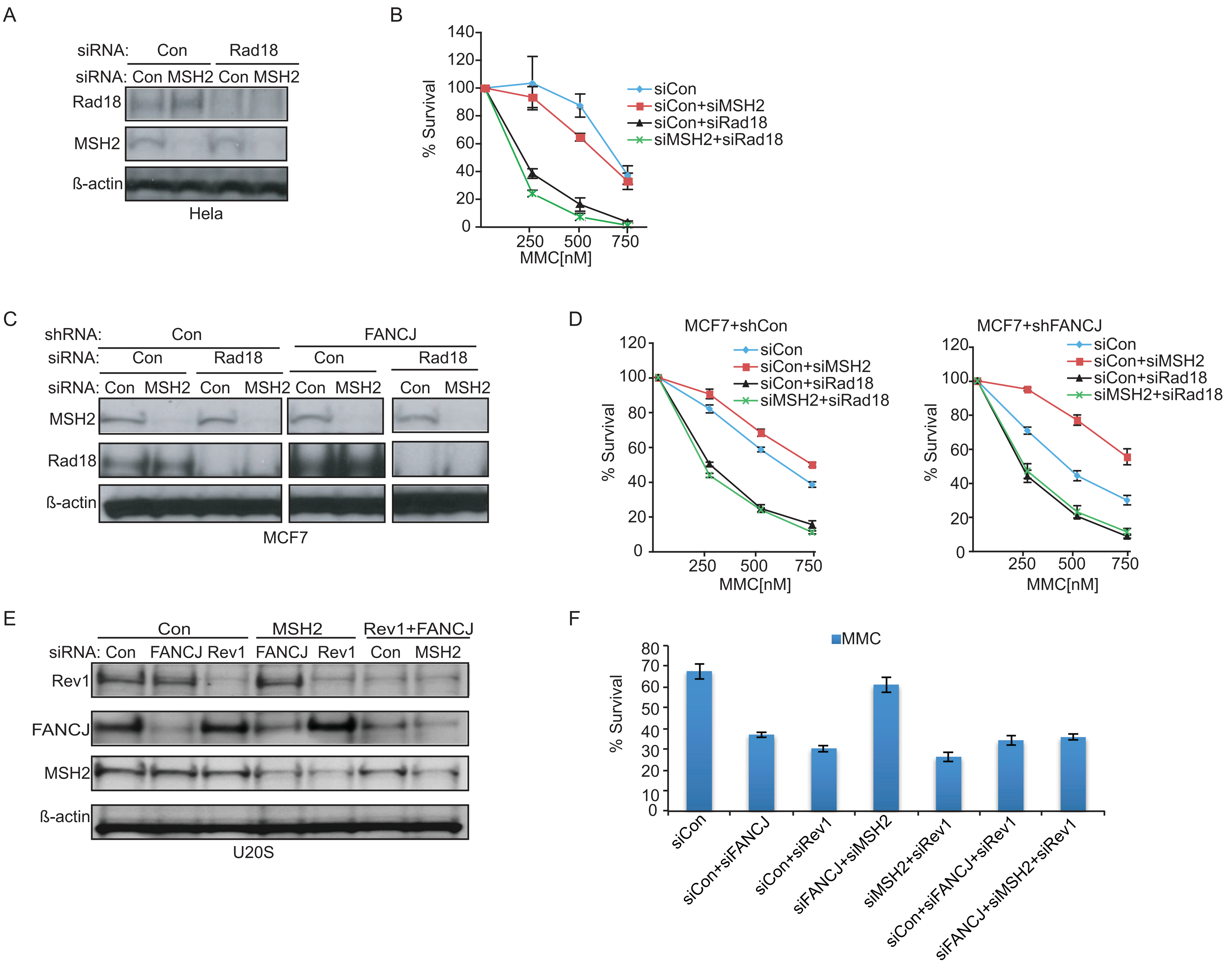

Supplementary Figure S5. Effects of MSH2 depletion on MMC sensitivity when Rad18 or Rev1 is also depleted. (A) Immunoblot analysis shows Rad18 and MSH2 expression in HeLa cells treated with indicated siRNAs. (B) Graph shows the percentage of viable cells 5 days after indicated dose of MMC. (C) Immunoblot analysis of MSH2 and Rad18 expression in MCF7 cells stably expressing shRNA to control or FANCJ treated with indicated siRNAs. (D) Graph shows the percentage of viable cells 5 days after indicated dose of MMC. (E) Immunoblot analysis of FANCJ, REV1 and MSH2 expression in U2OS cells treated with indicated siRNAs. β-actin is a loading control. (F) Graph shows the percentage of viable cells 5 days after 500nM MMC. Where shown, error bars represent standard deviations from three independent experiments.
