# Supplementary material for: Crosstalk between BRCA-Fanconi anemia and mismatch repair pathways prevents MSH2-dependent aberrant DNA damage responses
Source: EMBO J. 2014 Jun 26;33(15):1698–712. doi: 10.15252/embj.201387530 (PMC4194102; doi:10.15252/embj.201387530)
Supplement: Supplementary file 6 [file embj0033-1698-sd6.pdf]

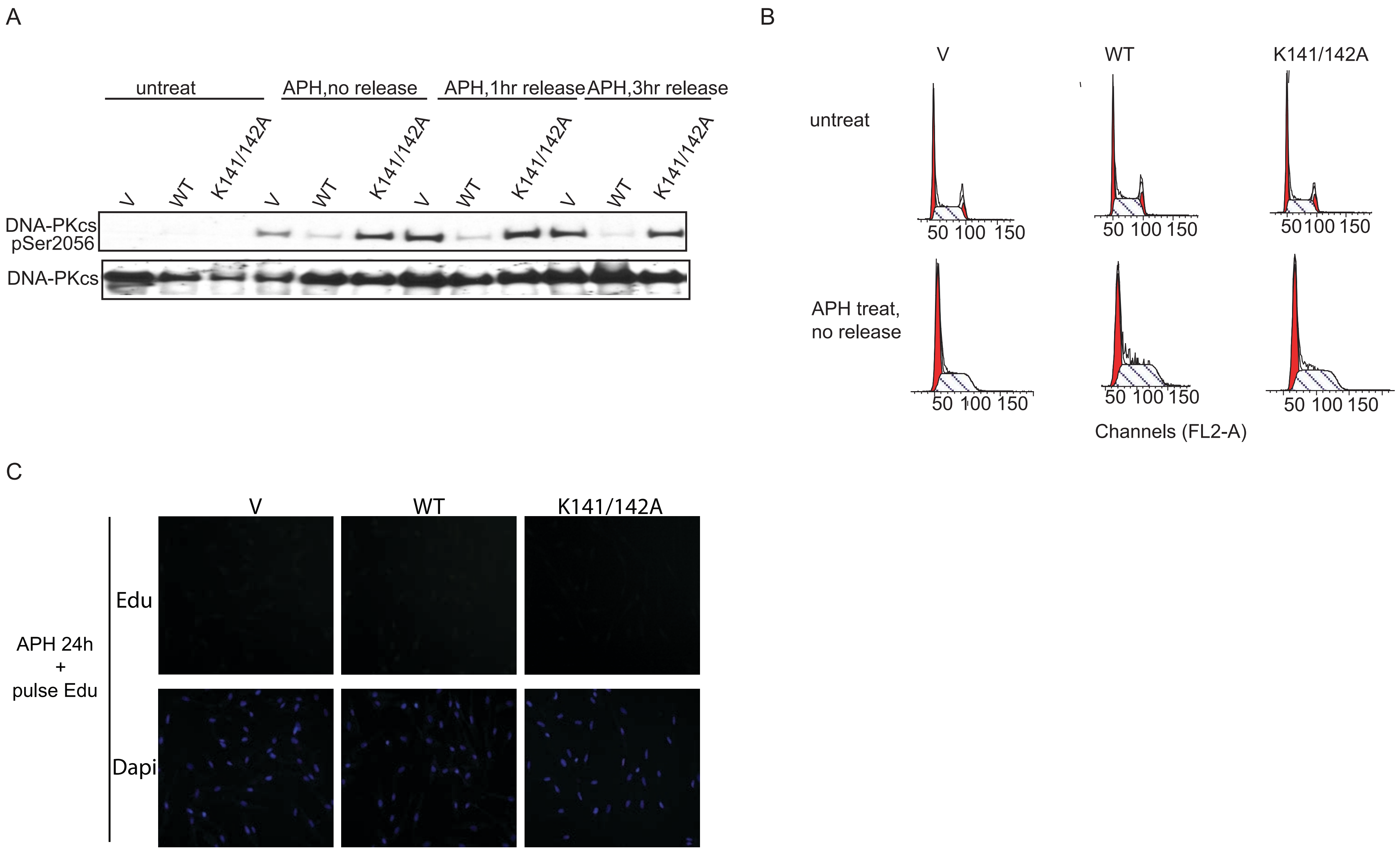

Supplementary Figure S6. FA-J cells lacking wild-type FANCJ have a pronounced activation of DNA-PKcs, but have a similar cell cycle and arrest follow-ing aphidicolin treatment as wild-type. (A) Immunoblot analysis of phosphorylation of DNA-PKcs in the FA-J cell lines before or after aphidicolin (APH) treatment and release. (B) FACS analysis shows that FA-J cell lines have a similar cell cycle profile after aphidocolin treatment. (C) Edu pulse and immunofloresence shows that APH (3ug/ml) treatment similarly blocks replication in FA-J cell lines.
