# Supplementary material for: Crosstalk between BRCA-Fanconi anemia and mismatch repair pathways prevents MSH2-dependent aberrant DNA damage responses
Source: EMBO J. 2014 Jun 26;33(15):1698–712. doi: 10.15252/embj.201387530 (PMC4194102; doi:10.15252/embj.201387530)
Supplement: Supplementary file 7 [file embj0033-1698-sd7.pdf]

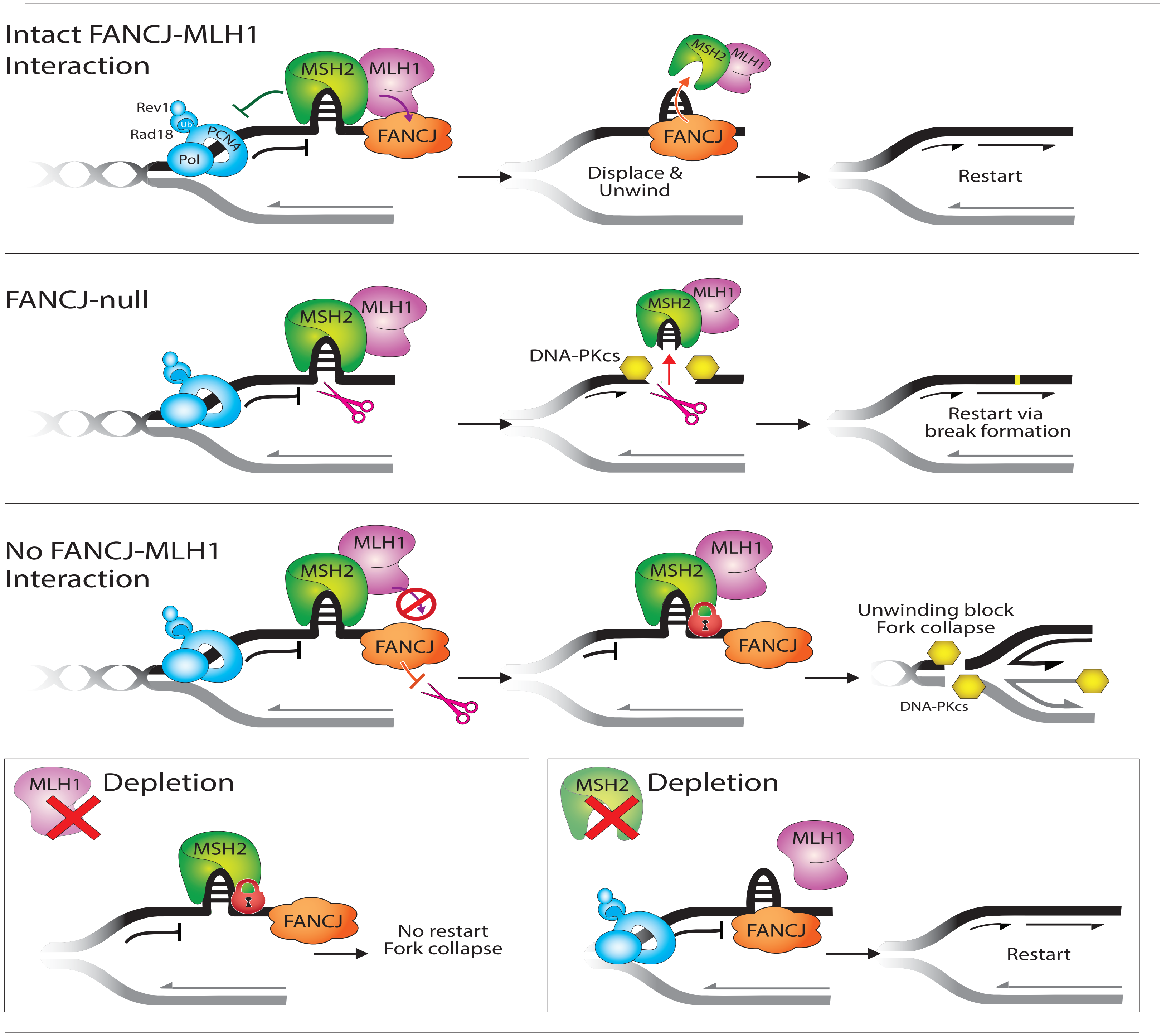

Supplementary Figure S7. Putative role of FANCJ-MLH1 interaction with MMR. Replication forks are blocked by barriers, such as secondary structures formed during replication stress as a consequence of MMC treatment. These barriers must be removed or by-passed for replication to proceed. **Intact FANCJ-MLH1 interaction:** Replication restart is coordinated with the MMR pathway to maintain genomic integrity. MSH2 heterodimers bind replication barriers, such as secondary structures (i.e. G4 quartets). FANCJ DNA helicase/translocase activity both displaces MSH2 and unwinds secondary structures to enable Rad18-dependent restart. **FANCJ null cells:** Nucleolytic processing removes secondary structures and induces breaks useful for restoring the restart of replication. MSH2 depletion does not alter this outcome because FANCJ is not available to unwind secondary structure. **No FANCJ-MLH1 interaction:** In the absence of MLH1 binding, FANCJ fails to displace MSH2 from secondary structure. MSH2 blocks restart and FANCJ blocks nucleolytic processing. Consequently, stalled forks collapse. MLH1 depletion will not rescue because MSH2 remains “locked” on replication barriers. MSH2 depletion will rescue because FANCJ gains ability to unwind replication barrier and replication resumes. MSH2 depletion also rescues FANCJ deficiency as long as some FANCJ is available to unwind the secondary structure.
