# Supplementary material for: Crosstalk between BRCA-Fanconi anemia and mismatch repair pathways prevents MSH2-dependent aberrant DNA damage responses
Source: EMBO J. 2014 Jun 26;33(15):1698–712. doi: 10.15252/embj.201387530 (PMC4194102; doi:10.15252/embj.201387530)
Supplement: Supplementary file 8 [file embj0033-1698-sd8.pdf]

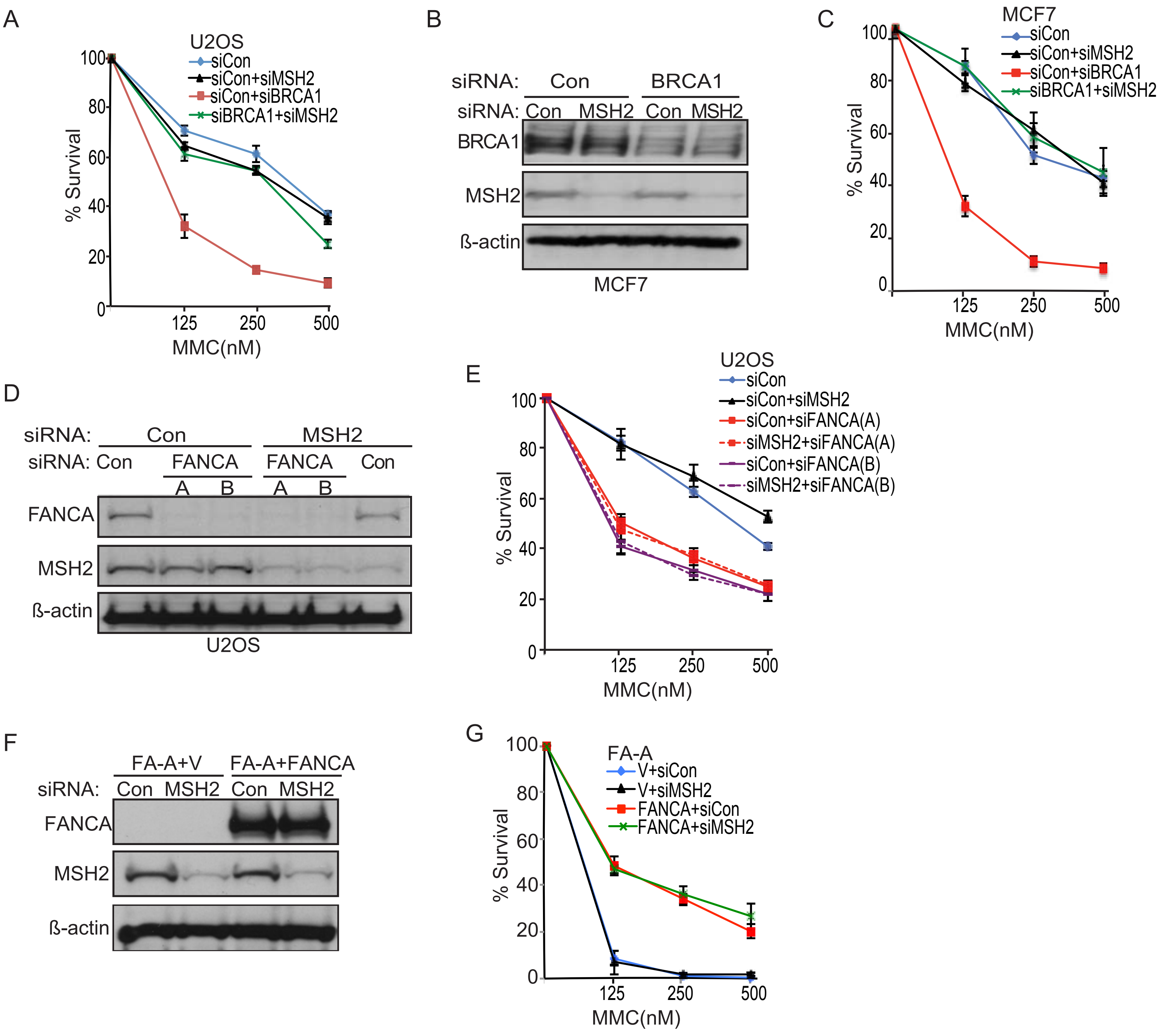

Supplementary Figure S8. MSH2 depletion suppresses MMC sensitivity in BRCA1-deficient cells, but not FANCA-deficient cells
(**A**) Graph shows the percentage of viable MCF7 cells 5 days after indicated dose of MMC and indicated siRNAs(immunoblot shown in Figure 6A). (**B**) Immunoblot analysis shows BRCA1 and MSH2 expression in MCF7 cells treated with indicated siRNAs. (**C**) Graph shows the percentage of viable cells 5 days after indicated dose of MMC. (**D**) Immunoblot analysis of FANCA and MSH2 expression in U20S cells treated with indicated siRNAs. (**E**) Graph shows the percentage of viable cells 5 days after indicated dose of MMC. (**F**) Immunoblot analysis of FANCA and MSH2 expression in FA-A(PD6914) cells stably expressing vectors(V) or FANCA that were also transfected with siRNAs to MSH2 or control. (**G**) Graph shows the percentage of viable cells 5 days after MMC. Where shown, error bars represent standard deviations from three independent experiments.
