# Supplementary material for: Crosstalk between BRCA-Fanconi anemia and mismatch repair pathways prevents MSH2-dependent aberrant DNA damage responses
Source: EMBO J. 2014 Jun 26;33(15):1698–712. doi: 10.15252/embj.201387530 (PMC4194102; doi:10.15252/embj.201387530)
Supplement: Supplementary file 9 [file embj0033-1698-sd9.pdf]

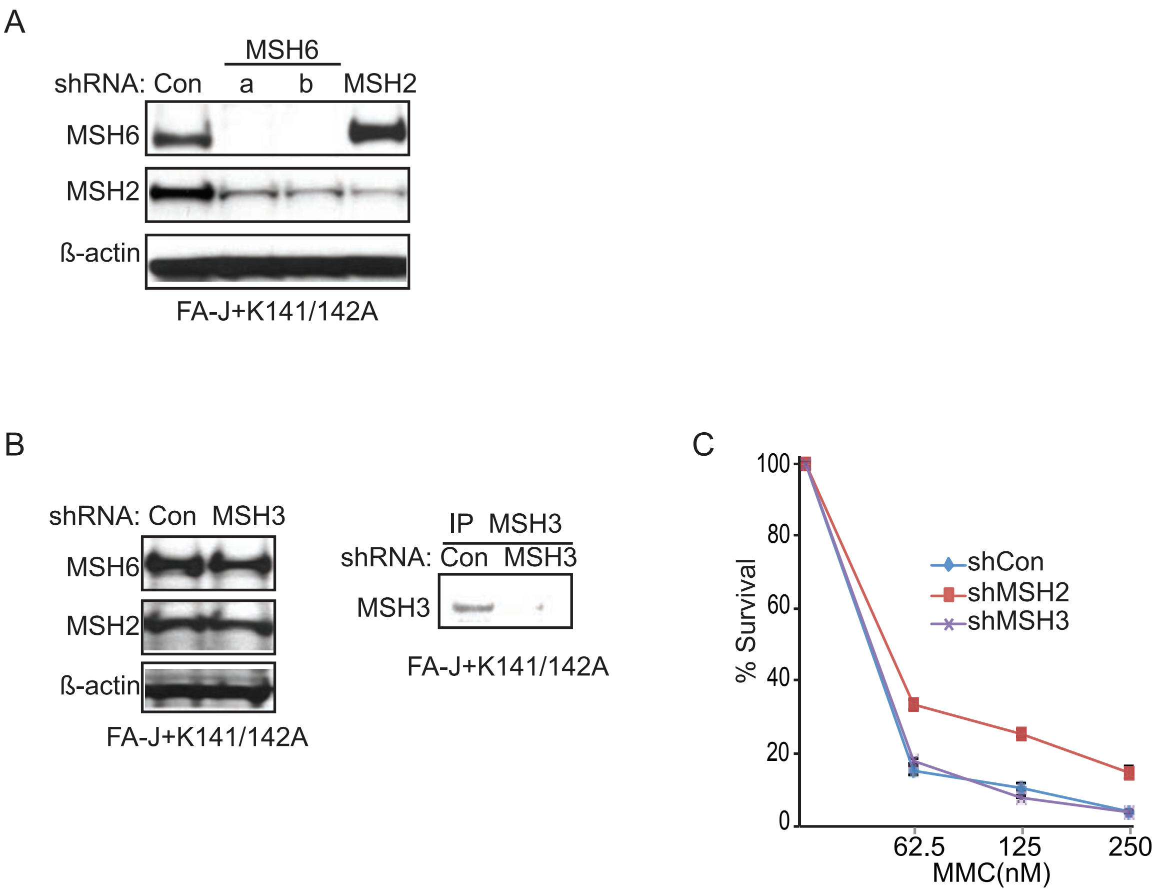

Supplementary Figure S9. MSH3 depletion does not suppress the MMC sensitivity in cells lacking the FANCJ-MLH1 interaction. (A) Immunoblot analysis of MSH6 and MSH2 expression in FA-J cells treated with indicated shRNAs shows that MSH6 shRNA also depletes MSH2. (B) Immunoblot analysis of MSH6, MSH2, and MSH3 expression in whole cell lysates or following an MSH3 immuno-precipitation from FA-J cell lysates treated with indicated shRNAs. (C) Graph shows percentage survival after indicated dose of MMC treatment. Where shown, error bars represent standard deviations from three independent experiments.
