# Supplementary material for: Crosstalk between BRCA-Fanconi anemia and mismatch repair pathways prevents MSH2-dependent aberrant DNA damage responses
Source: EMBO J. 2014 Jun 26;33(15):1698–712. doi: 10.15252/embj.201387530 (PMC4194102; doi:10.15252/embj.201387530)
Supplement: Supplementary file 10 [file embj0033-1698-sd10.pdf]

THE
EMBO
JOURNAL

Manuscript EMBO-2013-87530

Crosstalk between BRCA - Fanconi anemia and mismatch repair pathways prevents MSH2-dependent aberrant DNA damage responses

Min Peng, Jenny Xie, Anna Ucher, Janet Stavnezer, Sharon B. Cantor

Corresponding author: Sharon B. Cantor, UMASS Medical School

Review timeline:

Submission date:	27 November 2013
Editorial Decision:	23 December 2013
Revision received:	25 March 2014
Editorial Decision:	16 April 2014
Revision received:	16 May 2014
Accepted:	26 May 2014

Editor: Hartmut Vodermaier

Transaction Report:

(Note: With the exception of the correction of typographical or spelling errors that could be a source of ambiguity, letters and reports are not edited. The original formatting of letters and referee reports may not be reflected in this compilation.)

1st Editorial Decision 23 December 2013

Thank you for submitting your manuscript for consideration by The EMBO Journal. We have now received the comments of three expert referees which you will find copied below. As you will see, the reviewers find your results on rescue of MMC sensitivity of FANCJ-deficient cells by MSH2 loss of interest and potential importance. However, it is also apparent - especially from the comments of referees 1 and 3 - that they consider the work at this stage still somewhat too preliminary to offer the sort of conclusive insights that warrant publication in a broad general journal. The key issue in this respect is the currently mainly phenotypic nature of the analysis and the lack of conclusive mechanistic understanding, including in particular the absence of direct evidence for the proposed effects on replication fork restart. In addition, there are also substantial concerns with the decisiveness and technical quality of some of the results, with the potential to undermine the conclusions they are meant to support. These concerns, I am afraid to say, in our view preclude publication of the study in The EMBO Journal, at least in its present form.

However, we notice that an extended version of the study that addresses the key concerns of the reviewers could become a much more compelling candidate for our journal, and given the overall interest of the topic, I would therefore be willing to offer you an opportunity to respond to them by way of a revised manuscript. For such a revised version to warrant another round of review and to possibly proceed further towards publication, it will however be essential to directly address the molecular effects on replication forks in presented cellular models, and to further investigate the exact role of MSH2 and its complex partners. Furthermore, it will be important to improve the various technical concerns raised to the referees' satisfaction. On the other hand, I feel that more detailed and extensive mouse work (as requested by referee 3) would not be necessary within the scope of this study, pending adequate improvement of the other key issues.

I realize that satisfactorily addressing these various issues may not be trivial and will likely require a substantial further amount of time and experimental effort, and I would therefore also understand if you preferred to rather seek rapid publication without major changes elsewhere; however in light of the well-taken concerns raised by our referees, I feel that such improvement and mechanistic extension would really be essential for this study to become a sufficiently compelling candidate for a broad general journal such as The EMBO Journal. Should you decide to submit a revised version to our journal, please keep in mind that it is our policy to allow a single round of major revision only, and that it will therefore be essential to diligently and thoroughly answer to all the points raised by the reviewers at this stage (including those mentioned above where we consider further experiments beyond the scope of the revision). Please also bear in mind that your letter of response to the referees' comments will form part of the Review Process File, and will therefore be available online to the community. For more details on our Transparent Editorial Process, please visit our website: http://www.nature.com/emboj/about/process.html

We generally allow three months as standard revision time, and it is our policy that competing manuscripts published during this period will have no negative impact on our final assessment of your revised study. However, we request that you contact the editor as soon as possible upon publication of any related work, to discuss how to proceed. Should you foresee a problem in meeting this three-month deadline, please let us know in advance and we may be able to grant an extension.

Thank you again for the opportunity to consider this work for publication, and please do not hesitate to contact me in case you should have any additional question regarding this decision or the reports. I look forward to your revision.

REFEREE REPORTS:

Referee #1:

Peng and colleagues report on a suppression of lethality in FANCJ cells that specifically cannot interact with MLH1. This in turn, is suggested reveal a role for FANCJ-MLH1 in evicting MMR complexes (pivotally MSH2) that are proposed to recognise stalled forks and /or intermediates arising. A number of approaches, mainly phenotypic analysis are presented in support of this overall thesis. The work is of general interest, and could have significant impact on the field. However, there are quite a number of technical concerns that impact on the ability of the data to support the conclusions drawn. Moreover, there are several issues with the overall interpretation of the data that are inconsistent:

Technical issues:

- In Fig. 1 A: knockdown of MSH2 isn't particularly impressive. Moreover, the levels of FANCJ fluctuate wildly across the gel, and appear to be reduced by MLH1 and MSH2 depletion to a level comparable to that seen using the FANCJ siRNA.

- In Fig. 1B, is that data extrapolated form the survival curves presented in Suppl. Fig. 1A-C? If so, why not show the full curves in the main data as they are more informative? Also, in Fig. 1B, what is the significance asterisk on the siMSH2 siFANCJ compared to/with?

- Fig. 3B - It is necessary to see the original data from which these numbers were derived, these summary plots are difficult to judge.

- Fig. 3C - 'GFP' indicates what?

- Fig. 3D - The suggestion that pRNA and gH2AX are changed by MSH shRNA in this figure is not supported by the gels - there is no significant change.

- The data in Fig. 4 does not support the conclusions drawn. Simply looking at gH2AX and Rad51 at

a fixed time post MMC does not inform about the dynamics of these proteins, and any alterations. HR defects would be expected to manifest as a lack of resolution of these signals at later times points. Besides, in Fig. 3D the authors claim that shMSH2 in FA-J K141/152 cells alters gH2AX which is inconsistent to the data in 4B. Regarding Fig. 4C, are FA-J K141/152 cells Zeocin sensitive relative to WT, or are helicase-dead? if not, the experiment is uninformative. Also, to my knowledge the contribution of HR repair to Zeocin-induced DSBs is small. In Fig. 4E, the depletion of Rad18 is very poor, and causes concern for the conclusions drawn.

- Fig. 5C should be quantified.

- Fig. 5D - The changes in PKcs phosphorylation (which needs a loading control of total DNA-PKcs, as is 3D) between control and MSH2 depletion are not apparent to this reviewer. The same goes for RPA - although there is some decrease in pRPA the blot is poor (there is more pRPA in the untreated control pRPA lane than almost any other lane).

- Discussion - There is substantial discussion of the fate of replication forks in regard of the model presented. I would suggest that the study would be much more complete if this was directly assessed, to deal with the authors' thesis that MSH2 loss improves the ability of stalled forks to restart in FA cells.

Referee #2:

Comments on the MS: "MSH2 exacerbates the DNA damage response in Fanconi anemia deficient cells"

Peng M et al, 2013.

DNA interstrand crosslinks are repaired by at least 16 protein factors in the BRCA-FA pathway. At the cellular level, mutations which inactivate any of these genes, such as mutations in BRCA1 or FANCD2, cause a failure to repair damage caused by agents such as mitomycin C (MMC). A number of genetic interactions modify crosslink sensitivity in BRCA-FA mutant cells. Ablation of the NHEJ pathway has been shown to mitigate the genotoxic effects of interstand crosslinking in cells carrying mutations in some, but not all, BRCA-FA genes. FANCJ is an interesting member of the BRCA-FA family, which interacts with BRCA1 and, as the authors previously showed, the mismatch repair protein MLH1. Unlike most of the BRCA-FA proteins, it has no known role in upstream interstrand crosslink excision by the FA 'core complex', or in downstream homologous recombination events.

The manuscript shows that MSH2 depletion (but not MLH1 depletion) reduces MMC sensitivity, DNA damage signaling, G2 accumulation and replication arrest in FANCJ-knockdown cells. The effect of MSH depletion shown is remarkably strong. The observation of significant rescue of FANCJ phenotypes by MSH2 depletion strikes me as novel and interesting. There is a good amount of interesting data in this manuscript, and in terms of mechanism, the authors show by depletion of RAD18 that the effect of MSH2 is caused by interfering with RAD18-mediated replication fork restart.

The authors also provide data from double-knockout mice, which substantiate the possibility that targeting MSH2 can reverse symptoms of FA. In contrast to a previous report about FANCD2/Mlh1 dko mice (de Vrugt et al, 2009), FANCD2/Msh2 dko mice were born at close to Mendelian ratios. This manuscript therefore extends previous findings regarding the effect of targeting MMR in cases of FA and points to mechanistic differences in the effect of Mlh1 and Msh2 during ICL repair (possibilities which are covered in the discussion.). The mouse work definitely strengthens the findings, which otherwise rely a lot on cell lines and multiple shRNA treatments.

I had a few minor points:

1. 53BP1 is referred to as a 'NHEJ factor'. 'DNA damage response' factor or something similar would be more appropriate because 53BP1 is certainly not a canonical NHEJ factor and may not be

involved in NHEJ at all.
2. "As expected that FANCJK141/142A did not restore MMC resistance to FA-J cells as found for FANCDWT (Figure 2B) (Peng et al 2007)" - This language doesn't make sense- please re-write.
3. The word 'effect' is mis-spelled as 'affect' at two places in the MS.
4. Can the authors add further comment, based on their findings, about the potential advantages and disadvantages of targeting MSH2 as a therapeutic strategy in FA patients?

Referee #3:

Peng et al propose that the mismatch repair protein MSH2 somehow activates a detrimental repair pathway when the Fanconi anemia genes are absent. This is suggested to be a potential cause of the phenotype in some FA patients. While the links between mistmatch repair and the FA-BRCA pathway are of potential interest to readers of the EMBO J, this study lacks any real significant conclusions. The main experimental approach in the manuscript is to co-deplete/knockout various genes in the FA pathway and mismatch repair pathway and look at changes in sensitivity to various forms of DNA damage. A large number of interesting hypotheses are drawn in the discussion section, but these are not investigated in the experimental section.

For example, a conclusion is made that in "BRCA-FA deficient cells MSH2 loss improves the ability of stalled forks to restart, but does not elevate DNA repair". Firstly, this conclusion is based only on FANCJ and FANCD2 cells - which have previously been shown to have the most severe FA phenotype and therefore results may not be extendable to other FA subtypes (eg FANCA, that accounts for >65% of FA). Secondly, there are no primary experiments related to fork restart or DNA repair - only indirect readouts of different forms of foci formation. Thirdly, why are FANCJ-null cells not rescued by MSH2 depletion (sup fig 2), only FANCJ-siRNA?

Other aspects of the manuscript are also lacking in in-depth investigation. For example, in results presented in figure 6, FANCD2-/- MEFs are rescued by MSH2-/-. What was the point of using mice when the authors have only investigated the same events seen in immortalised cell lines? Does MSH2-/- rescue other phenotypes of FANCD2-/- mice like increased microphthalmia, perinatal lethality, and epithelial cancers?

And is the same phenotype seen with MSH3 or MSH6 knockdown? MSH2 dimerizes with these proteins in its MMR function, but they're barely mentioned in this paper! This is a big omission that needs to be investigated.

This study is interesting, but further experiments testing the hypotheses outlined in the discussion section are required to elevate this manuscript from more than a purely observational study.

Final comment: A clearer title is required. "Fanconi anemia deficient cells" doesn't make sense. You could use "FANCD2-deficient cells" or "Fanconi anemia cells". A title suggestion would be "MSH2 exacerbates the DNA damage response in the absence of the Fanconi anemia pathway". The running title is also about MLH1 for some reason!

1st Revision - authors' response 25 March 2014

We thank the reviewers for their thoughtful and helpful critiques of our manuscript entitled "MSH2 exacerbates the DNA damage response in Fanconi anemia deficient cells". We note that reviewers voiced some concerns about technical issues and mechanistic understanding relating to replication fork restart, which we have addressed in detail and comprehensively. These clearly strengthen the manuscript as outlined in red below in which we respond to each of the specific points raised by the reviewers:

In response to Reviewer #1:

1) In Fig. 1 A: knockdown of MSH2 isn't particularly impressive. Moreover, the levels of FANCJ fluctuate wildly across the gel, and appear to be reduced by MLH1 and MSH2 depletion to a level

comparable to that seen using the FANCJ siRNA.

In response, we agree that this immunoblot could be misleading and have made several changes to improve interpretation. First, we provide a new immunoblot, which is more representative of the type of depletion obtained by MSH2 siRNAs. Second, we improve the order and labeling of the figure so that it is clear which lanes have single or dual siRNAs and therefore would be expected to have reduced FANCJ expression.

2) In Fig. 1B, is that data extrapolated form the survival curves presented in Suppl. Fig. 1A-C? If so, why not show the full curves in the main data as they are more informative? Also, in Fig. 1B, what is the significance asterisk on the siMSH2 siFANCJ compared to/with?

In response, we now include the full survival curves in Figure 1B. The original bar graph representation is in revised Supplementary Figure 1B so that a direct comparison can be made. In addition, we include the required information in the figure to understand the asterisk comparison.

3) Fig. 3B - It is necessary to see the original data from which these numbers were derived, these summary plots are difficult to judge.

As requested, the original data are included in revised Figure 3B.

4) Fig. 3C - 'GFP' indicates what?

*In response, we clarify here and in the revised manuscript (page 8) that green fluorescent protein (*GFP*) is expressed simultaneously from the same vector as the* small hairpin RNA, *facilitating the identification of infected cells.*

5) Fig. 3D - The suggestion that pRNA and gH2AX are changed by MSH shRNA in this figure is not supported by the gels - there is no significant change.

We agree that the affect of MSH2 depletion on phosphorylation of RPA and g-H2AX is subtle. In order to increase the sensitivity of the detection, we now instead include analysis of chromatin fractions in revised Figure 4C and D, more clearly showing that MSH2 depletion suppresses phosphorylation of RPA. The reduction in phosphorylation of g-H2AX is less apparent but also reproducible and supported by immunofluorescence experiments (new Supplementary Figure 4B).

6) The data in Fig. 4 does not support the conclusions drawn. Simply looking at gH2AX and Rad51 at a fixed time post MMC does not inform about the dynamics of these proteins, and any alterations. HR defects would be expected to manifest as a lack of resolution of these signals at later times points. Besides, in Fig. 3D the authors claim that shMSH2 in FA-J K141/142 cells alters gH2AX which is inconsistent to the data in 4B. Regarding Fig. 4C, are FA-J K141/142 cells Zeocin sensitive relative to WT, or are helicase-dead? if not, the experiment is uninformative. Also, to my knowledge the contribution of HR repair to Zeocin-induced DSBs is small.

In response to these points, first, we agree that analysis of Rad51 foci at a single time-point post-damage is only informative if robust changes are detected, and in this case they are not. As suggested, we include the analysis of Rad51 foci at several time points post MMC (new Supplementary Figure 4A). As shown here and in original Figure 4B, we did not detect a change in Rad51 foci between control and MSH2 depleted cells that also had clear signs of MMC-induced g-H2AX foci. Second, we clarify that we do not claim that g-H2AX in Figure 4B is the same in the presence or absence of MSH2. Instead, Rad51 foci were analyzed only from cells that were also positive for g-H2AX. This was done to avoid the inclusion of cells that did not undergo a DNA damage response. The revised figure axis more clearly reflects this point. Finally in response to the concern that experiments with zeocin are uninformative and that zeocin generates breaks not processed significantly by HR, we agree and have removed this figure. We also removed the CPT data because FA-J cells, similar to other FA patient cells, are not very sensitive to agents that induce DNA double strand breaks and it is difficult to assess if MSH2 depletion rescues by survival assays. Instead, to better appreciate if MSH2 depletion alters HR, we analyzed MMC-induced Rad51 chromatin loading by immunoblot (revised Figure 4C). Similar to the immunofluorescence experiments, we did not detect measurable changes in the chromatin loading of Rad51 when MSH2

is depleted. However, as noted above, analysis of chromatin fractions clearly show reduced phosphorylation of DNA-PKcs and RPA as a consequence of MSH2 depletion (revised Figure 4D)(page 8-9). This more extensive analysis supports our conclusion that MSH2 depletion does not enhance the initiation or resolution of Rad51-based repair.

7) In Fig. 4E, the depletion of Rad18 is very poor, and causes concern for the conclusions drawn.

In response to the concern about Rad18 depletion, we performed densitometry on this immunoblot. Similar to our other experiments in which we attempt to deplete Rad18 in FA-J cells in dual depletion assays, we achieved ~50 percent reduction in Rad18 expression. Despite several attempts with distinct reagents, we have not been able to achieve greater Rad18 suppression, as achieved in HeLa or MCF7 cells (Supplementary Figure 5A and C).

8) Fig. 5C should be quantified.

In the revised manuscript, the data are quantified and shown in revised Figure 6D.

9) Fig. 5D - The changes in PKcs phosphorylation (which needs a loading control of total DNA-PKcs, as is 3D) between control and MSH2 depletion are not apparent to this reviewer. The same goes for RPA - although there is some decrease in pRPA the blot is poor (there is more pRPA in the untreated control pRPA lane than almost any other lane).

We addressed this concern by adding the requested loading control of total DNA-PKcs to the revised Figures including 3D, 4D, and 6E. Furthermore, we replaced original Figure 5D with a new image of the chromatin bound DNA-PKcs, which more clearly depicts the reduced phosphorylation achieved by MSH2 depletion (Figure 6E). In the chromatin fraction of MSH2 depleted cells, the reduction in RPA phosphorylation is also detected but remains modest and therefore was not included.

10) Discussion - There is substantial discussion of the fate of replication forks in regard of the model presented. I would suggest that the study would be much more complete if this was directly assessed, to deal with the authors' thesis that MSH2 loss improves the ability of stalled forks to restart in FA cells.

In response, we include additional data in which we analyze the effect of MSH2 depletion on the ability of replication forks to restart replication after release from the DNA polymerase inhibitor aphidicolin. First, we find that similar to treatment with MMC, vector and $FANCJ^{K141/142A}$ *as compared to wild-type FA-J cells have enhanced phosphorylation of DNA-PKcs (New Figure 5A and Supplementary Figure 6A). Second, we find that following aphidicolin release,* $FANCJ^{K141/142A}$ *cells, similar to vector FA-J cells fail to restart replication as measured by Edu incorporation (new Figure 5B and C). Third, FACs analysis reveals that* $FANCJ^{K141/142A}$ *FA-J cells have a severe replication restart defect, worse than vector cells. The* $FANCJ^{K141/142A}$ *FA-J cells take more than 72h to reach a 4N DNA content as compared to vector FA-J cells that take 24h (new Figure 5D). Finally, we found that MSH2 depletion alleviates this restart defect (new Figure 5E). Collectively these findings support the hypothesis that MSH2 interferes with replication restart in cells lacking the FANCJ-MLH1 interaction and we have added these points to the results section (page 9-10), describe in the Discussion (page 13 and 14) and note in the abstract.*

In response to Reviewer #2:

1) 53BP1 is referred to as a 'NHEJ factor'. 'DNA damage response' factor or something similar would be more appropriate because 53BP1 is certainly not a canonical NHEJ factor and may not be involved in NHEJ at all.

53BP1 has been shown to inhibit DNA end resection at DNA breaks, thereby promoting NHEJ and inhibiting HR (Bunting et al., Cell 2010). Thus, in this context, we refer to 53BP1 as a NHEJ factor.

2) "As expected that FANCJK141/142A did not restore MMC resistance to FA-J cells as found for FANCDWT (Figure 2B) (Peng et al 2007)" - This language doesn't make sense- please re-write.

As suggested, in the revised manuscript we rewrote the text, which now states, "as expected, FA-J cells complemented with FANCJ$^{K141/142A}$ remained sensitive to MMC, whereas FA-J cells complemented with wild-type FANCJ have enhanced resistance" (page 6).

3) The word 'effect' is mis-spelled as 'affect' at two places in the MS.

In the revised manuscript the mistake was fixed.

4) Can the authors add further comment, based on their findings, about the potential advantages and disadvantages of targeting MSH2 as a therapeutic strategy in FA patients?

As requested, further comment was added to the last paragraph in the revised discussion (page 15).

In response to Reviewer #3:

Peng et al propose that the mismatch repair protein MSH2 somehow activates a detrimental repair pathway when the Fanconi anemia genes are absent. This is suggested to be a potential cause of the phenotype in some FA patients. While the links between mismatch repair and the FA-BRCA pathway are of potential interest to readers of the EMBO J, this study lacks any real significant conclusions. The main experimental approach in the manuscript is to co-deplete/knockout various genes in the FA pathway and mismatch repair pathway and look at changes in sensitivity to various forms of DNA damage. A large number of interesting hypotheses are drawn in the discussion section, but these are not investigated in the experimental section.

1) For example, a conclusion is made that in "BRCA-FA deficient cells MSH2 loss improves the ability of stalled forks to restart, but does not elevate DNA repair". Firstly, this conclusion is based only on FANCJ and FANCD2 cells - which have previously been shown to have the most severe FA phenotype and therefore results may not be extendable to other FA subtypes (eg FANCA, that accounts for >65% of FA).

In response to this point, we agree and clarify in the revised Discussion that our findings are restricted to FANCJ and FANCD2, and that it will be important to address if MSH2 loss will rescue other FA cell lines (page 12).

2) Secondly, there are no primary experiments related to fork restart or DNA repair - only indirect readouts of different forms of foci formation.

In response to this point, we provide additional experiments show in new Figure 5. Specifically, we analyzed replication restart following release from the DNA polymerase inhibitor aphidicolin in a series of experiments including immunoblot, Edu incorporation, and FACs analysis. As described above, we find that cells expressing the FANCJ mutant that lacks MLH1 binding have a severe replication restart defect that is rescued by MSH2 depletion (Figure 5E).

3) Thirdly, why are FANCJ-null cells not rescued by MSH2 depletion (sup fig 2), only FANCJ-siRNA?

This is a reasonable question that can be explained by the fact that rescue requires that cells have some FANCJ helicase activity. Indeed, MSH2 depletion rescues cells that have FANCJ helicase activity (FANCJ$^{K141/142A}$ FA-J cells), but not cells without FANCJ helicase activity (vector or FANCJ^{K52R} FA-J cells). Rescue is also achieved in FANCJ siRNA depleted cells perhaps because residual FANCJ is sufficient to support its helicase function. This is further clarified in the Results and Discussion section (page 7 and 15).

4) Other aspects of the manuscript are also lacking in in-depth investigation. For example, in results presented in figure 6, FANCD2-/- MEFs are rescued by MSH2-/-. What was the point of using mice when the authors have only investigated the same events seen in immortalised cell lines? Does MSH2-/- rescue other phenotypes of FANCD2-/- mice like increased microphthalmia, perinatal lethality, and epithelial cancers?

We agree that the RNAi approach strongly supports a role for MSH2 in mediating defects in FA-D2 and FA-J cells lacking the FANCJ-MLH1 interaction. However, mouse genetics provide a rigorous system to confirm these results in normal cells. We also concur that additional experiments will be important to better understand how or if MSH2 contributes to the unique features of a Fancd2 mouse, but we believe that these experiments are beyond the scope of the current manuscript.

5) And is the same phenotype seen with MSH3 or MSH6 knockdown? MSH2 dimerizes with these proteins in its MMR function, but they're barely mentioned in this paper! This is a big omission that needs to be investigated.

We agree with this reviewer that it is worth pursuing the role of MSH3 and MSH6. In this regard, we have attempted to address how MSH3 or MSH6 depletion affects MMC resistance in FANCJ-deficient and $FANCJ^{K141/142A}$ *FA-J cells. However, immunoblot analysis demonstrates that MSH6 depletion also reduces MSH2 expression (new Supplementary Figure 8). This reduction in MSH2 expression by MSH6 RNAi is not unique to our study (Mohni et al., J of Virology 2011). Thus, it was not possible to interpret how MSH6 depletion affected MMC sensitivity. In contrast, MSH3 depletion did not reduce MSH2 or MSH6 protein levels (Supplementary Figure 8B). However, detection of MSH3 expression in FA-J cells was difficult and required MSH3 immunoprecipitation preceding immunoblot analysis (Supplementary Figure 8B). Notably, MSH3 depletion also did not enhance MMC resistance in* $FANCJ^{K141/142A}$ *FA-J cells, whereas MSH2 deletion does (Supplementary Figure 8C). Thus, the MSH3-MSH2 complex may not contribute to MMC sensitivity and by process of elimination MSH6-MSH2 complex may contribute. These points were added to the Discussion (page 14).*

6) This study is interesting, but further experiments testing the hypotheses outlined in the discussion section are required to elevate this manuscript from more than a purely observational study.

As suggested by the reviewer, in the revised manuscript, we include additional experiments (new Figure 5) and the mechanistic understanding that MSH2 interferes with replication restart.

7) Final comment: A clearer title is required. "Fanconi anemia deficient cells" doesn't make sense. You could use "FANCD2-deficient cells" or "Fanconi anemia cells". A title suggestion would be "MSH2 exacerbates the DNA damage response in the absence of the Fanconi anemia pathway". The running title is also about MLH1 for some reason!

As suggested by this reviewer, the title was changed to "MSH2 exacerbates the DNA damage response in the absence of the Fanconi anemia pathway". We also changed the running title to " MSH2 is toxic in FA".

In summary, we have responded to the referees' criticisms and believe our revised manuscript is greatly improved. It provides evidence that BRCA-FA and MMR pathways converge to ensure ICL processing without corruption by unproductive MSH2 activities that stalls replication forks. We believe that it will be of broad interest to the readership of *EMBO Journal*.

2nd Editorial Decision 16 April 2014

Thank you for submitting your revised manuscript on MSH2-FANCJ interactions for our consideration. The study has now once more been seen by the three original reviewers, whose comments are copied below. As you will see, although the referees acknowledge the overall improvements in this revised manuscript, they still retain substantial reservations regarding publication at the present stage. These concerns include some persisting technical issues, but also the overriding criticism that key conclusions of the paper, as proposed prominently in title and abstract, are still not decisively supported by the results presented in the manuscript.

As emphasized in my first decision letter, we generally only allow for one single round of experimental revision; in order to avoid lengthy revision processes extended over multiple round of

incremental improvements. In the present case, I would in light of the interest of the topic and the improvements already made (especially with regard to possible replication restart roles) remain open to considering an exceptional second revision of this study, in case you should be able to (a) address the technical points still retained by the referees (regarding the knockdown efficiency problems, I agree with referee 2 that the mouse data serve as important validation in this respect), and (b) to add conclusive further data on MSH2 knockdown effects in conditions of BRCA1/2 and FA core complex deficiency or downregulation (as explained by referee 3) that would justify the generally stated conclusions and broaden the overall significance of the presented findings.

At this stage, I am therefore returning the manuscript to you once again, hoping you will be able to obtain the decisive data required for making this study a strong candidate for an EMBO Journal article during an additional round of major revision. Please note that this will however have to be the final revision round for this manuscript at our journal. Should you have any question in this regard, please do not hesitate to contact me and I'd be happy to discuss them.

REFEREE REPORTS:

Referee #1:

Peng et a have improved this MS, but several technical issues and other concerns are still present:

Fig. 1A and elsewhere - that the knockdowns are not highly efficient does raise concerns about the robustness of the conclusions drawn. I do accept that this could be tricky to improve on.

Fig, 4C and D - The chromatin fractions are not well controlled for. It is always desirable to see the non-chromatin fractions alongside (with appropriate controls for enrichment of each sub-cellular fraction) these to reassure that there is no contamination from cytoplasm etc. Also, because the changes reported on are modest we need absolute confidence of loading, and the RPA and PKcs which are used to monitor this (Mcm7 as used in panel 4C is much better, though) in 4D are very heavily loaded and overexposed making even-ness of loading hard to assess. Overall, I am concerned that the quantitative aspects of this part of the paper lack robustness.

Suppl. Fig. 4 - This important time-course figure requires proper quantification. Statements regarding gH2AX (the foci of which look somewhat atypical to me) are not convincing based upon visual inspection (bottom of page 8).

Referee #2:

In this revised manuscript, the authors have been responsive to the initial comments, and made a number of improvements to the data. I agreed with many of the other reviewers' comments, which highlighted some weaknesses with the original manuscript. One issue is the widespread use of knockdown approaches, which are never 100% efficient. In this case some of the knockdowns look incomplete- for example the RAD18 knockdown in Figure 4. Nonetheless, I am persuaded that there is a phenotype here, supported by several assays, which is of some interest.

I disagree with Reviewer #3 that using knockout mice (Figure 6 in original manuscript; Figure 7 in current version) is a problem considering that most of the work is done with cell lines. I believe that the mouse genetics is more reliable than the knockdown experiments. Without the mouse data, I would find the manuscript to be less convincing. I tend to agree with the authors that the mouse work has value and should be included.

With these types of studies, mechanistic detail makes a lot of difference. The authors do offer some mechanistic insight in terms of replication fork stalling, and an apparent involvement of RAD18. I'm not left with a totally clear picture of what MSH2 is doing to corrupt DNA repair in the absence of FANC factors, but the authors certainly raise some interesting possibilities in the discussion section.

I have a question about the title, which was changed to "MSH2 exacerbates the DNA damage response in the absence of the Fanconi anemia pathway". 'Exacerbate' isn't really the right term, and the 'Fanconi anemia pathway' is not really absent- just selected components of it. I would suggest something like: "MSH2 interferes with replication-fork restart in cells with deficiencies in FANCJ and FANCD2".

The abstract should be re-written too. In its current form, it doesn't even hint at a mechanism for how MSH2 depletion causes rescue of pathological phenotypes arising in FANC-deficient cells treated with MMC.

Referee #3:

The resubmitted manuscript of Peng et al has addressed several of the technical issues raised by myself and the other reviewers and is now a stronger manuscript. But I am still not satisfied that the findings are of general relevance to Fanconi anemia or familial breast cancer. I am still uncomfortable with the constant references to the "BRCA-FA pathway" throughout the manuscript when it is clear that the interaction with mismatch repair is only demonstrated for FANCJ and FANCD2.
While the discussion has been changed, the title, abstract, introduction and several other paragraphs refer to the "BRCA-FA" pathway. If it is really this pathway then it should be demonstrated that MSH2 knockdown interacts with loss of BRCA1 or BRCA2 and at least one component of the core complex at the very least.

MSH2 knockdown should be possible in the matched HCC1937 cells for BRCA1, and several matched FANCA of FANCC knockout pairs (eg available from the FA research fund) for the core complex. The reason I stress this point is because it is demonstrated in figure 1 that FANCJ (and FANCD2) cells are NOT the same as other FA cells with respect to rescue by DNA-PKcs inhibitors or 53BP1 knockdown. An alternative would be a refocus on links between FANCJ and MMR rather than the "BRCA-FA pathway" to prevent unexplored conclusions being drawn by readers.

I still believe that the use of mice in figure 7 adds no additional information to the manuscript and the mice should have instead been used to analyse phenotypes rather than just as a source of cells. The concluding hypothesis about "progression to cancer" and "useful for therapy" would have been tight and medically relevant. But given the work involved in an extended study it probably is "beyond scope".

One other thing I noticed is in figure 3F, the average aberrations per metaphase is 9 for control siRNA and 4 for MSH2 siRNA in FA-J cells, but the examples provided in figure 3 have two and zero aberrations highlighted. More representative examples should be provided.

2nd Revision - authors' response 16 May 2014

© European Molecular Biology Organization

University of Massachusetts
UMASS Medical School

May 16, 2014

We thank the reviewers for their thoughtful and helpful critiques of our manuscript tracking number now entitled "MSH2 interferes with recovery from replication stress in cells lacking crosstalk between BRCA-Fanconi anemia and mismatch repair pathways". We comprehensively addressed the remaining technical issues and the criticism that key conclusions of the paper are not supported by the results in the manuscript. In particular, we add further data demonstrating that MSH2 depletion improves survival of BRCA1 deficient cells in response to MMC. In contrast, however we find that MSH2 depletion does not rescue the MMC sensitivity of FANCA deficient cells. These additional data suggest that MSH2 contributes to an aberrant DNA damage response in some, but not all BRCA-FA cells. Whereas FANCJ, BRCA1 and FANCD2 interact with MMR proteins, an interaction between FANCA and MMR has not been reported. Thus, the rescue by MSH2 depletion is not universal to all BRCA-FA cells and instead could stratify by BRCA-FA proteins that interact with MMR. Based on these additional data, the title, abstract, and text were altered.

In response to Reviewer #1:

Fig. 1A and elsewhere - that the knockdowns are not highly efficient does raise concerns about the robustness of the conclusions drawn. I do accept that this could be tricky to improve on. We agree with the reviewer that greater depletion could be very difficult to achieve; however, it is satisfying that the depletion obtained for FANCJ generates MMC sensitivity and the depletion obtained for MMR proteins generates MNU resistance (Figure 1B and 6B and Supplementary Figure 1A). These outcomes suggest the depletion obtained does interfere with function of these proteins.

Fig, 4C and D - The chromatin fractions are not well controlled for. It is always desirable

to see the non-chromatin fractions alongside (with appropriate controls for enrichment of each sub-cellular fraction) these to reassure that there is no contamination from cytoplasm etc. Also, because the changes reported on are modest we need absolute confidence of loading, and the RPA and PKcs which are used to monitor this (Mcm7 as used in panel 4C is much better, though) in 4D are very heavily loaded and overexposed making even-ness of loading hard to assess. Overall, I am concerned that the quantitative aspects of this part of the paper lack robustness. We clarify that MSH2 depletion reduces phosphorylation of DNA-PKcs when analyzed in total cell lysates or in a nuclear/chromatin fraction. However, its phosphorylation was more robust in the chromatin and therefore its reduction more apparent following MSH2 depletion. As not to confuse the reader, that we were measuring changes in localization (cytoplasmic vs. chromatin), but rather phosphorylation of DNA-Pkcs, the soluble fraction is not included in Figure 4C or D. The designation chromatin fraction was also removed and instead the method of lysis is described in the figure legend. Total DNA-Pkcs and RPA are ideal for interpreting changes in how these proteins are phosphorylated. Thus, the overexposed RPA loading control was replaced with one that is not overexposed and interpretable.

Suppl. Fig. 4 - This important time-course figure requires proper quantification. Statements regarding gH2AX (the foci of which look somewhat atypical to me) are not convincing based upon visual inspection (bottom of page 8). The requested quantification of γ-H2AX is included for Supplementary Figure 4C. The current magnification of 20X was used so that a large field of cells could be observed, but as viewed at 60X as shown in Supplementary Figure 4A the γ-H2AX are focal as expected.

In response to Reviewer #2:

In this revised manuscript, the authors have been responsive to the initial comments, and made a number of improvements to the data. I agreed with many of the other reviewers' comments, which highlighted some weaknesses with the original manuscript. One issue is the widespread use of knockdown approaches, which are never 100% efficient. In this case some of the knockdowns look incomplete- for example the RAD18 knockdown in Figure 4. Nonetheless, I am persuaded that there is a phenotype here, supported by several assays, which is of some interest.

I disagree with Reviewer #3 that using knockout mice (Figure 6 in original manuscript; Figure 7 in current version) is a problem considering that most of the work is done with cell lines. I believe that the mouse genetics is more reliable than the knockdown experiments. Without the mouse data, I would find the manuscript to be less convincing. I tend to agree with the authors that the mouse work has value and should be included.

With these types of studies, mechanistic detail makes a lot of difference. The authors do offer some mechanistic insight in terms of replication fork stalling, and an apparent involvement of RAD18. I'm not left with a totally clear picture of what MSH2 is doing to corrupt DNA repair in the absence of FANC factors, but the authors certainly raise some interesting possibilities in the discussion section.

I have a question about the title, which was changed to "MSH2 exacerbates the DNA damage response in the absence of the Fanconi anemia pathway". 'Exacerbate' isn't really the right term, and the 'Fanconi anemia pathway' is not really absent- just selected components of it. I would suggest something like: "MSH2 interferes with replication-fork restart in cells with deficiencies in FANCJ and FANCD2".
In response, we include potential titles such as: MSH2 interferes with recovery from replication stress in cells lacking crosstalk between BRCA-FA and mismatch repair pathways, or MSH2 contributes to the aberrant DNA damage response and replication recovery defects in cells lacking crosstalk between BRCA-Fanconi anemia and mismatch repair pathways.

The abstract should be re-written too. In its current form, it doesn't even hint at a mechanism for how MSH2 depletion causes rescue of pathological phenotypes arising in FANC-deficient cells treated with MMC.
In response, the abstract was rewritten and includes that rescue by MSH2 depletion is Rad18 dependent.

In response to Reviewer #3:

The resubmitted manuscript of Peng et al has addressed several of the technical issues raised by myself and the other reviewers and is now a stronger manuscript. But I am still not satisfied that the findings are of general relevance to Fanconi anemia or familial breast cancer. I am still uncomfortable with the constant references to the "BRCA-FA pathway" throughout the manuscript when it is clear that the interaction with mismatch repair is only demonstrated for FANCJ and FANCD2. While the discussion has been changed, the title, abstract, introduction and several other paragraphs refer to the "BRCA-FA" pathway. If it is really this pathway then it should be demonstrated that MSH2 knockdown interacts with loss of BRCA1 or BRCA2 and at least one component of the core complex at the very least. MSH2 knockdown should be possible in the matched HCC1937 cells for BRCA1, and several matched FANCA of FANCC knockout pairs (eg available from the FA research fund) for the core complex. The reason I stress this point is because it is demonstrated in figure 1 that FANCJ (and FANCD2) cells are NOT the same as other FA cells with respect to rescue by DNA-PKcs inhibitors or 53BP1 knockdown. An alternative would be a refocus on links between FANCJ and MMR rather than the "BRCA-FA pathway" to prevent unexplored conclusions being drawn by readers.

In response to the concern that a rescue outcome is restricted to the loss of a distinct set of BRCA-FA factors, we have further examined this issue. Indeed, we find that MSH2 depletion suppresses the ICL-sensitivity of BRCA1 deficient, but not FANCA deficient cells (revised Figure 6A and B and new Supplementary Figure 8). These new data raise the interesting possibility that rescue will be restricted to BRCA-FA factors that normally form interactions with MMR proteins. Currently this list includes FANCJ, BRCA1, FANCD2 and SLX4. Alternatively, rescue could be restricted to BRCA-FA factors that are not essential for bypass pathways or that function “downstream” in the

BRCA-FA pathway. Defining if MSH2 depletion rescues loss of BRCA-FA proteins linked to a particular function, will be an important future direction. To prevent unexplored conclusions being drawn, we have modified the title, abstract, and discussion.

I still believe that the use of mice in figure 7 adds no additional information to the manuscript and the mice should have instead been used to analyse phenotypes rather than just as a source of cells. The concluding hypothesis about "progression to cancer" and "useful for therapy" would have been tight and medically relevant. But given the work involved in an extended study it probably is "beyond scope".
We agree that these are important extensions of the work that should be explored.

One other thing I noticed is in figure 3F, the average aberrations per metaphase is 9 for control siRNA and 4 for MSH2 siRNA in FA-J cells, but the examples provided in figure 3 have two and zero aberrations highlighted. More representative examples should be provided. In response to this point, the quantitation is from 50 metaphases and capturing individual images with this exact ratio 9 vs. 4 was challenging. We chose the current images because the left panel clearly demonstrates the type of lesions found in response to MMC that were suppressed by MSH2 depletion, as shown in the right panel. We clarify this point in the revised figure legend.

In summary, we have responded to the additional referees' concerns and believe this revised manuscript will be of broad interest to the readership of *EMBO Journal*.
